# White matter and nigral alterations in multiple system atrophy-parkinsonian type

**DOI:** 10.1038/s41531-021-00236-0

**Published:** 2021-10-29

**Authors:** Takashi Ogawa, Taku Hatano, Koji Kamagata, Christina Andica, Haruka Takeshige-Amano, Wataru Uchida, Daiki Kamiyama, Yasushi Shimo, Genko Oyama, Atsushi Umemura, Hirokazu Iwamuro, Masanobu Ito, Masaaki Hori, Shigeki Aoki, Nobutaka Hattori

**Affiliations:** 1 Department of Neurology, Faculty of Medicine, Juntendo University, Tokyo, Japan; 2 Department of Radiology, Faculty of Medicine, Juntendo University, Tokyo, Japan; 3 Department of Neurology, Juntendo University Nerima Hospital, Tokyo, Japan; 4 Department of Neurosurgery, Faculty of Medicine, Juntendo University, Tokyo, Japan; 5 Department of Psychiatry, Faculty of Medicine Juntendo University, Tokyo, Japan; 6 Department of Radiology, Toho University Omori Medical Center, Tokyo, Japan

**Keywords:** Predictive markers, Parkinson's disease

## Abstract

Multiple system atrophy (MSA) is classified into two main types: parkinsonian and cerebellar ataxia with oligodendrogliopathy. We examined microstructural alterations in the white matter and the substantia nigra pars compacta (SNc) of patients with MSA of parkinsonian type (MSA-P) using multishell diffusion magnetic resonance imaging (dMRI) and myelin sensitive imaging techniques. Age- and sex-matched patients with MSA-P (*n* = 21, *n* = 10 first and second cohorts, respectively), Parkinson’s disease patients (*n* = 19, 17), and healthy controls (*n* = 20, 24) were enrolled. Magnetization transfer saturation imaging (MT-sat) and dMRI were obtained using 3-T MRI. Measurements obtained from diffusion tensor imaging (DTI), free-water elimination DTI, neurite orientation dispersion and density imaging (NODDI), and MT-sat were compared between groups. Tract-based spatial statistics analysis revealed differences in diffuse white matter alterations in the free-water fractional volume, myelin volume fraction, and intracellular volume fraction between the patients with MSA-P and healthy controls, whereas free-water and MT-sat differences were limited to the middle cerebellar peduncle in comparison with those with Parkinson’s disease. Region-of-interest analysis of white matter and SNc revealed significant differences in the middle and inferior cerebellar peduncle, pontine crossing tract, corticospinal tract, and SNc between the MSA-P and healthy controls and/or Parkinson’s disease patients. Our results shed light on alterations to brain microstructure in MSA.

## Introduction

Multiple system atrophy (MSA) is a progressive neurodegenerative disorder characterized by parkinsonism that responds poorly to levodopa, as well as autonomic failure and cerebellar ataxia^1^. Clinically, MSA is divided into parkinsonian (MSA-P) and cerebellar types. Pathologically, glial cytoplasmic inclusions (GCIs), which are associated with α-synuclein aggregation, appear in the oligodendrocytes of patients with MSA, predominantly in the pontine nucleus, olivary nucleus, cerebellum, substantia nigra, striatum, and white matter^2^. The accumulation of these GCIs causes chronic neuroinflammation^3^.

Several imaging studies of MSA patients have reported the utility of diffusion tensor imaging (DTI) for discriminating MSA from Parkinson’s disease (PD)^4–6^. In recent years, more advanced diffusion magnetic resonance imaging (MRI) methods, such as neurite orientation dispersion and density imaging (NODDI)^7^, have been developed to capture more detailed alterations in brain microstructure. NODDI allows the parameters of intracellular volume fraction (ICVF; indicating neurite [axon and dendrites] density based on intracellular diffusion), orientation dispersion index (ODI; indicating neurite dispersion), and isotropic volume fraction (ISOVF; indicating the volume fraction of isotropic diffusion, such as that which occurs in cerebrospinal fluid) to be obtained^7,8^. Furthermore, the fractional volume of free water (FW) reflects isotropic water diffusion in the interstitial extraneuronal space^9,10^, and increases in response to neuroinflammation, axonal injury, and demyelination^11^. FW-eliminated DTI (FWE-DTI), which involves the elimination of alterations from FW, can provide measures of FW-corrected fractional anisotropy (FA_T_), FW-corrected mean diffusivity (MD_T_), FW-corrected axial diffusivity (AD_T_), and FW-corrected radial diffusivity (RD_T_), which are more specific to tissue alterations and neurodegeneration than the corresponding measures obtained from conventional DTI^12–15^. Recently, FW studies in MSA patients have been reported^16–18^, but they focused on the basal ganglia and substantia nigra only. Furthermore, myelin-sensitive imaging techniques, such as magnetization transfer saturation (MT-sat) imaging, also exist and are suitable for the evaluation of white matter demyelination. These allow a myelin volume fraction (MVF) to be obtained, with low values indicating demyelination (Supplementary Table 1)^19^.

In the present study, we hypothesized that advanced diffusion MRI and myelin-sensitive imaging would allow us to detect more specific pathologies—such as neuroinflammation, neurodegeneration, and demyelination—than conventional MRI in the white matter of patients with MSA-P, which may be useful for understanding the pathological mechanisms of MSA-P^3^. To evaluate this hypothesis, we used DTI, NODDI, FWE-DTI, and MT-sat imaging to investigate the white matter and substantia nigra of healthy controls (HCs), patients with PD, and patients with MSA-P.

## Results

### Demographic and clinical features of participants

We analyzed a second cohort to corroborate the results obtained in the first cohort. Table 1 shows the demographic data, clinical features, and MRI findings of both the first and second cohorts. Both cohorts had similar demographics, but the MRI findings of a pontine or middle cerebellar peduncle sign were less pronounced in the second cohort. There were no significant differences in age or sex between any of the groups, or in the levodopa equivalent daily dose (LEDD) or the presence of rapid eye movement sleep behavior disorder (RBD) between MSA-P and PD patients. The MSA-P patients had a significantly longer (*P* < 0.05) disease duration, higher Hoehn & Yahr (HY) stage, and higher Movement Disorder Society Unified PD Rating Scale (MDS-UPDRS) part 3 score (Japanese-translated version) than the PD patients. The Unified MSA Rating Scale (UMSARS) part 2 was only assessed in the MSA-P patients. T2-weighted imaging (WI) in patients with MSA-P showed the pontine cross sign, the vertical hyperintensity line in the pons, cerebellar atrophy, hyperintensity of the middle cerebellar peduncle (MCP), and the putaminal slit (Table 1).

**Table 1:** Demographics, clinical features, and MRI findings in the various subject groups.

	First cohort			Second cohort		
	HCs	PD	MSA-P	HCs	PD	MSA-P
Participants, *n*	*n* = 20	*n* = 19	*n* = 21	*n* = 24	*n* = 17	*n* = 10
Age, years	62.8 ± 4.7	63.1 ± 8.1	62.5 ± 11.7	65.8 ± 6.5	63.2 ± 10.2	65.3 ± 9.5
Male, % (*n*)	40.0% (8/20)	35.0% (7/20)	40.0% (8/20)	54.2% (13/24)	58.8% (10/17)	70.0% (7/10)
Disease duration, years	NA	5.2 ± 1.6	3.4 ± 1.7*	NA	4.7 ± 2.1	3.3 ± 0.95*
Hoehn & Yahr stage	NA	2.1 ± 0.8	3.2 ± 0.7**	NA	1.9 ± 0.7	3.0 ± 0.8**
MDS-UPDRS part 3	NA	15.3 ± 11.3	40.1 ± 16.0**	NA	18.8 ± 13.3	56.1 ± 15.3**
UMSARS part 2	NA	NA	21.2 ± 8.7	NA	NA	27.9 ± 7.3
Levodopa equivalent daily dose, mg	NA	677.0 ± 362.6	658.0 ± 310.0	NA	662.5 ± 509.1	859.1 ± 480.2
RBD present, % (*n*)	NA	36.8% (7/19)	47.6% (10/21)	NA	23.5% (4/17)	40.0% (4/10)
**MRI findings**						
Pontine cross sing, % (*n*)	NA	0% (0/19)	9.5% (2/21)	NA	0% (0/17)	0.0% (0/10)
vertical hyperintensity line in the pons, % (*n*)	NA	0% (0/19)	42.1% (8/21)*	NA	0% (0/17)	10.0% (1/10)
Cerebellar atrophy, % (*n*)	NA	0% (0/19)	71.4% (15/21)**	NA	0% (0/17)	80.0% (8/10)**
Middle cerebellar peduncle-sign, % (*n*)	NA	0% (0/19)	4.8% (1/21)	NA	0% (0/17)	0.0% (0/10)
Putaminal slit, % (*n*)	NA	0% (0/19)	85.7% (18/21)**	NA	0% (0/17)	90.0% (9/10)**

*HCs* healthy controls, *MDS-UPDRS* Movement Disorder Society Unified Parkinson’s Disease Rating Scale, *MSA-P* multiple system atrophy-Parkinsonian type, *NA* not applicable, *PD* Parkinson’s disease, *RBD* rapid eye movement-sleep behavior disorder, *UMSARA part 2* Unified Multiple System Atrophy Rating Scale.

*P < *0.05*; MSA-P vs. PD, **P < *0.01*, MSA-P vs. PD.

### Voxel-wise tract-based spatial statistics (TBSS) analysis

**Fig. 1:**
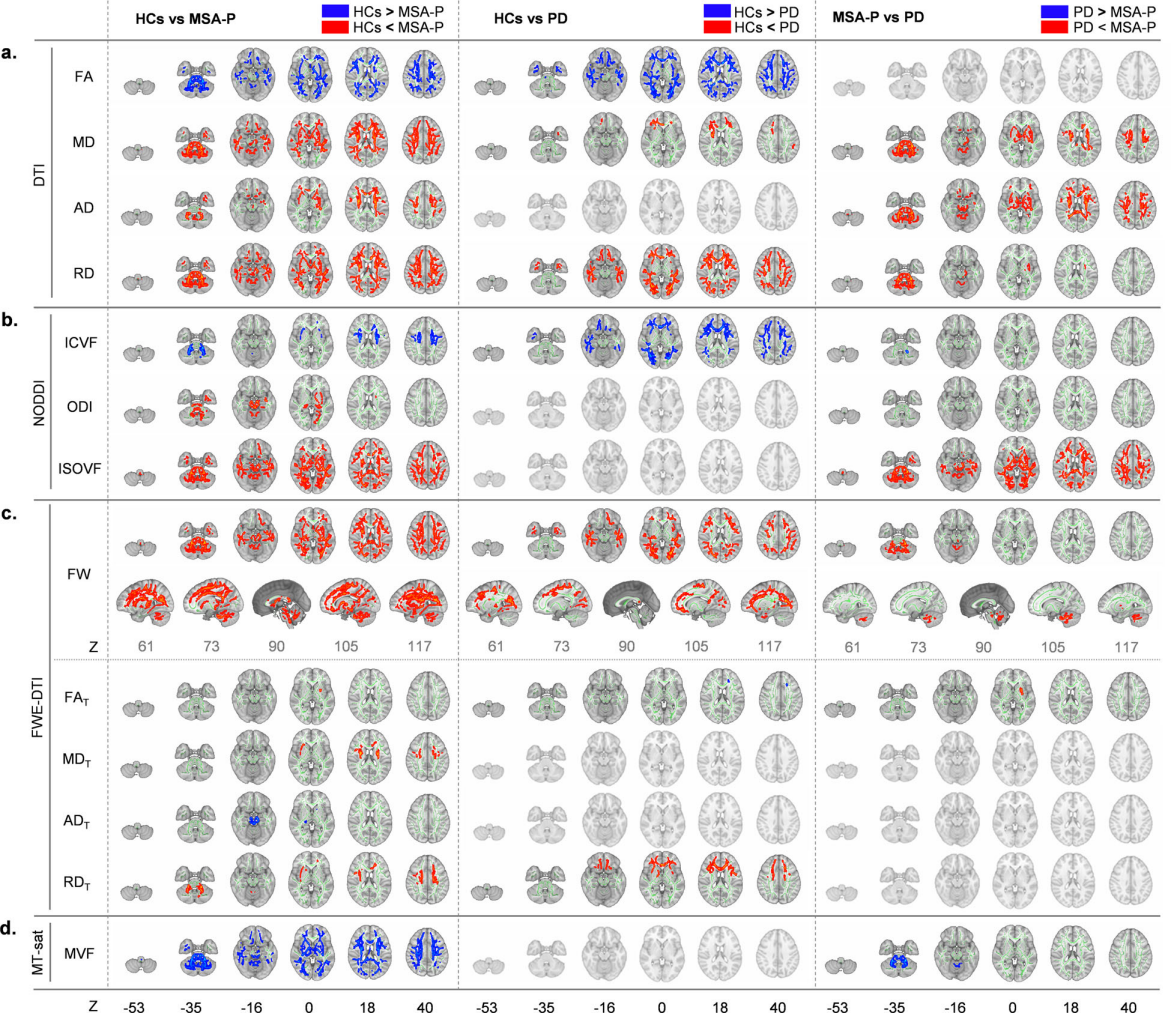
Comparison of DTI, NODDI, FWE-DTI, and MT-sat. Diffusion tensor imaging (DTI, fractional anisotropy [FA], mean diffusivity [MD], axial diffusivity [AD], and radial diffusivity [RD]) (**a**), neurite orientation dispersion and density imaging (NODDI, intracellular volume fraction [ICVF], orientation dispersion index [ODI], and isotropic volume fraction [ISOVF]) (**b**), free-water elimination DTI (FWE-DTI, FW, free water-corrected FA [FA_T_], free water-corrected MD [MD_T_], free water-corrected AD [AD_T_], and free water-corrected RD [RD_T_]) (**c**), and magnetization transfer-saturation (MT-sat, myelin volume fraction [MVF]) (**d**) indices. Tract-based spatial statistics (TBSS) analyses showed that MSA-P patients had significantly (*P* < 0.05, FWE-corrected) lower (blue/light blue voxels) FA, ICVF, MVF, and AD_T_, and higher (red-yellow voxels) MD, AD, RD, ODI, ISOVF, FW, FA_T_, MD_T_, and RD_T_ than healthy controls, and significantly higher (blue/light blue voxels) MD, RD, FW, and RD_T_, and lower (red-yellow voxels) FA, ICVF, and FA_T_ than Parkinson’s disease patients. MSA-P patients showed lower (blue/light blue voxels) ICVF and MVF, and higher (red-yellow voxels) MD, AD, RD, ODI, ISOVF, FW, and FA_T_, compared with Parkinson’s disease patients. There were no significant differences in MVF between the groups (see Supplementary Table 2). The TBSS results were very similar in the second cohort (see Supplementary Table 3). The skeleton is presented in green. To aid visualization, the results are thickened using the fill script implemented in the FMRIB Software Library.

TBSS analyses were performed using the acquired MRI data, but the MVF was not calculated for the second cohort; these subjects did not undergo MT-sat because of a difference in the imaging protocol (see Methods). We compared DTI (FA, MD, AD, and RD), NODDI (ICVF, ODI, and ISOVF), FWE-DTI (FW, FA_T_, MD_T_, AD_T_, and RD_T_), and MT-sat (MVF) indices between HCs, PD patients, and MSA-P patients (Fig. 1, first cohort). Details of the anatomical regions, peak *t* values, and peak Montreal Neurological Institute (MNI) coordinates of the significant clusters are shown in Supplementary Tables 2 and 3 (first and second cohorts, respectively).

In the first cohort, DTI analyses revealed diffuse differences between the groups in the cerebral and cerebellar white matter and the brainstem (Fig. 1a, Supplementary Table 2). Specifically, the MSA-P patients had significantly (family-wise error-corrected *P* value < 0.05) lower FA and higher MD, AD, and RD compared with the HCs, while the PD patients had significantly lower FA and higher MD and RD compared with the HCs. Furthermore, the MSA-P patients had significantly higher MD, AD, and RD than the PD patients. The PD patients showed no significant differences in FA and AD compared with either the HCs or MSA-P patients. In the second cohort, very similar results (i.e., higher values) were obtained for MD, AD, and RD in the MSA-P patients compared with the HCs and PD patients (Supplementary Table 3).

In the first cohort, differences in the NODDI indices of ICVF or ODI in the MSA-P patients (Fig. 1b, Supplementary Table 2) were largely limited to the brainstem, in contrast to the diffuse differences that were obtained with DTI. In diffuse white matter regions, there was significantly lower ICVF and higher ODI and ISOVF in the MSA-P patients compared with the HCs, and significantly lower ICVF in the PD patients compared with the HCs. Furthermore, the MSA-P patients had significantly lower ICVF in the MCP, significantly higher ODI in the external capsule (EC), and diffuse regions of significantly higher ISOVF compared with the PD patients. In the second cohort, very similar results were obtained, with the MSA-P patients showing diffuse regions of lower ICVF and higher ISOVF compared with the HCs, as well as significantly higher ODI in the EC (Supplementary Table 3).

In the first cohort, FWE-DTI analyses of MSA-P patients revealed significant differences in the brainstem, including in the MCP (Fig. 1c). Specifically, there were diffuse regions of significantly higher FW, a narrow area of significantly lower AD_T_, and a narrow area of significantly higher FA_T_, MD_T_, and RD_T_ in the MSA-P patients compared with the HCs, as well as significantly lower FA_T_ and higher FW and RD_T_ in the PD patients compared with the HCs. Furthermore, the MSA-P patients had significantly higher FW and FA_T_ compared with the PD patients. Very similar results for FW, MD_T_, and RD_T_ were obtained in the second cohort when comparing the MSA-P patients with HCs, as well as for FW in the PD patients compared with HCs (Supplementary Table 3).

MT-sat analyses, which were only performed on the first cohort, revealed lower MVF in the MSA-P patients compared with the HCs and PD patients (Fig. 1d). There were no differences between the HCs and PD patients.

### Region-of-interest (ROI) analysis

**Fig. 2:**
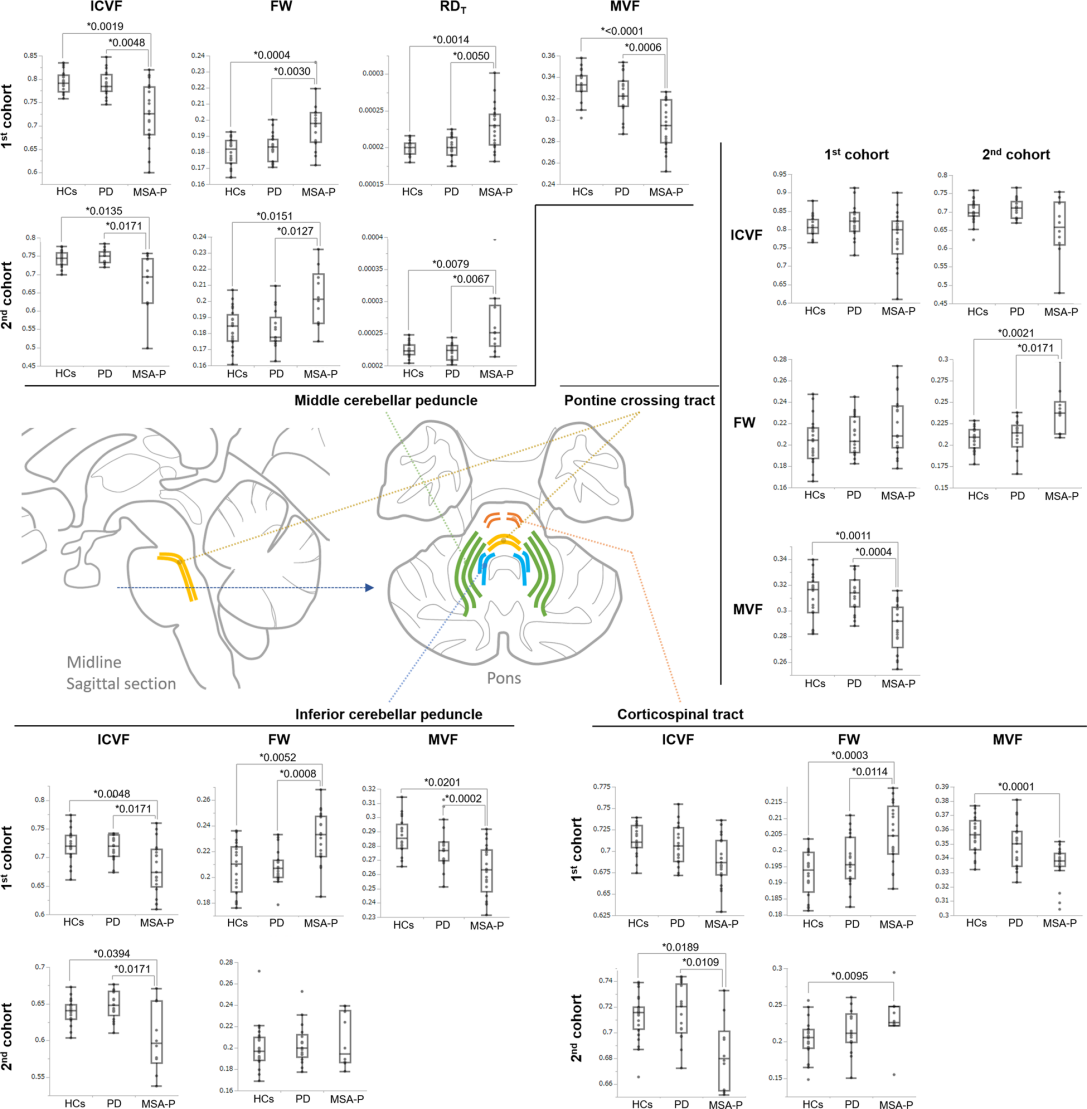
Results of the white matter ROI analysis and schematic imaging of significant regions. Within the white matter, there were significantly lower (*P* < 0.05, false discovery rate [FDR]-corrected) ICVF and MVF values, and significantly higher FW or RD_T_ in the middle cerebellar peduncle (MCP; green line in schematic) and inferior cerebellar peduncle (ICP; light blue line) in MSA-P patients compared with HCs and PDs. The MVF values in the pontine crossing tract (yellow line) were significantly lower in MSA-P patients compared with HCs and PD, whereas FW in the corticospinal tract (CST; light red line) was significantly higher in MSA-P patients. In the second cohort of white matter, very similar results of first cohort were observed in the four regions of MCP, ICP, PCT, and CST. The differences from the first cohort were the absence of significant differences in FW in the ICP (although a non-significant trend remained), significantly higher FW in the PCT, and significantly lower ICVF in the CST of MSA-P patients compared with HCs and PD patients. In the boxplot, the center line represents the median, the box represents the third quartile, and the whiskers represent the maximum and minimum values. FW free water, ICVF intracellular volume fraction, MVF myelin volume fraction, pSN posterior part of SNc, RD_T_ free water-corrected radial diffusivity, aSN anterior part of SNc, SNc substantia nigra pars compacta.

ROI analysis was performed based on the regions in a white matter atlas; 30 ROIs were analyzed to comprehensively investigate MSA-P-specific white matter changes (see Methods for more details). In the first cohort, the white matter microstructures of MSA-P patients were significantly different to those of HCs and PD patients according to the NODDI index of ICVF, MT-sat index of MVF, FWE-DTI indices of FW, and RD_T_ in five regions after false discovery rate (FDR) correction. In MSA-P patients, there was significantly higher ICVF in the MCP and inferior cerebellar peduncle (ICP), and significantly lower MVF in the MCP, ICP, and pontine crossing tract (PCT). There was also significantly higher FW in the MCP, ICP, corticospinal tract (CST), and fornix/stria terminalis in MSA-P patients (Fig. 2, Supplementary Table 4). In the second cohort, very similar results were observed in the MCP, ICP, PCT, and CST. However, in contrast to the first cohort, there was an absence of significant differences in FW in the fornix/stria terminalis and ICP (although a non-significant trend remained), significantly higher FW in the PCT, and significantly lower ICVF in the CST of MSA-P patients compared with HCs and PD patients (Fig. 2, Supplementary Table 4).

**Fig. 3:**
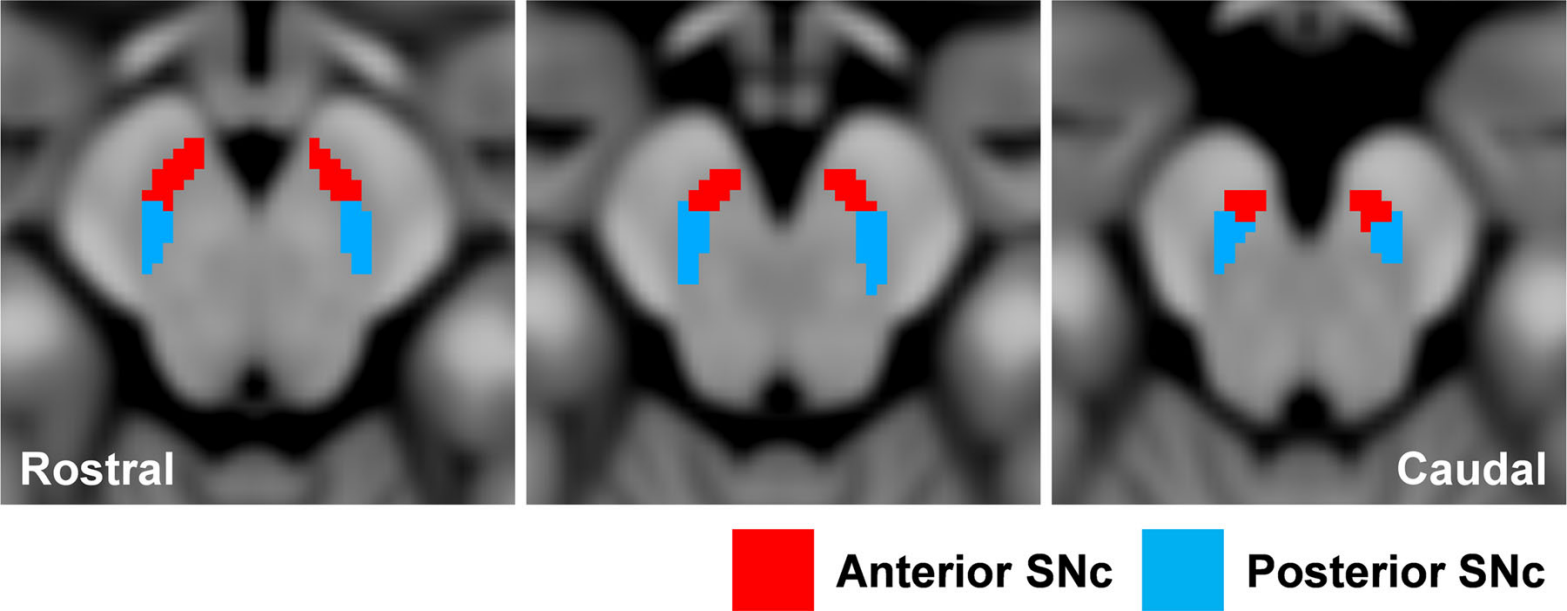
The anterior and posterior ROI of substantia nigra pars compacta. The SNc ROI was automatically created with using automated anatomical atlas 3rd version (AAL3). The anterior substantia nigra (aSN; blue) and the posterior substantia nigra (pSN; red) were manually divided in the middle of each section of SNc atlas (see Methods). ROI region-of-interest, SNc substantia nigra pars compacta.

**Fig. 4:**
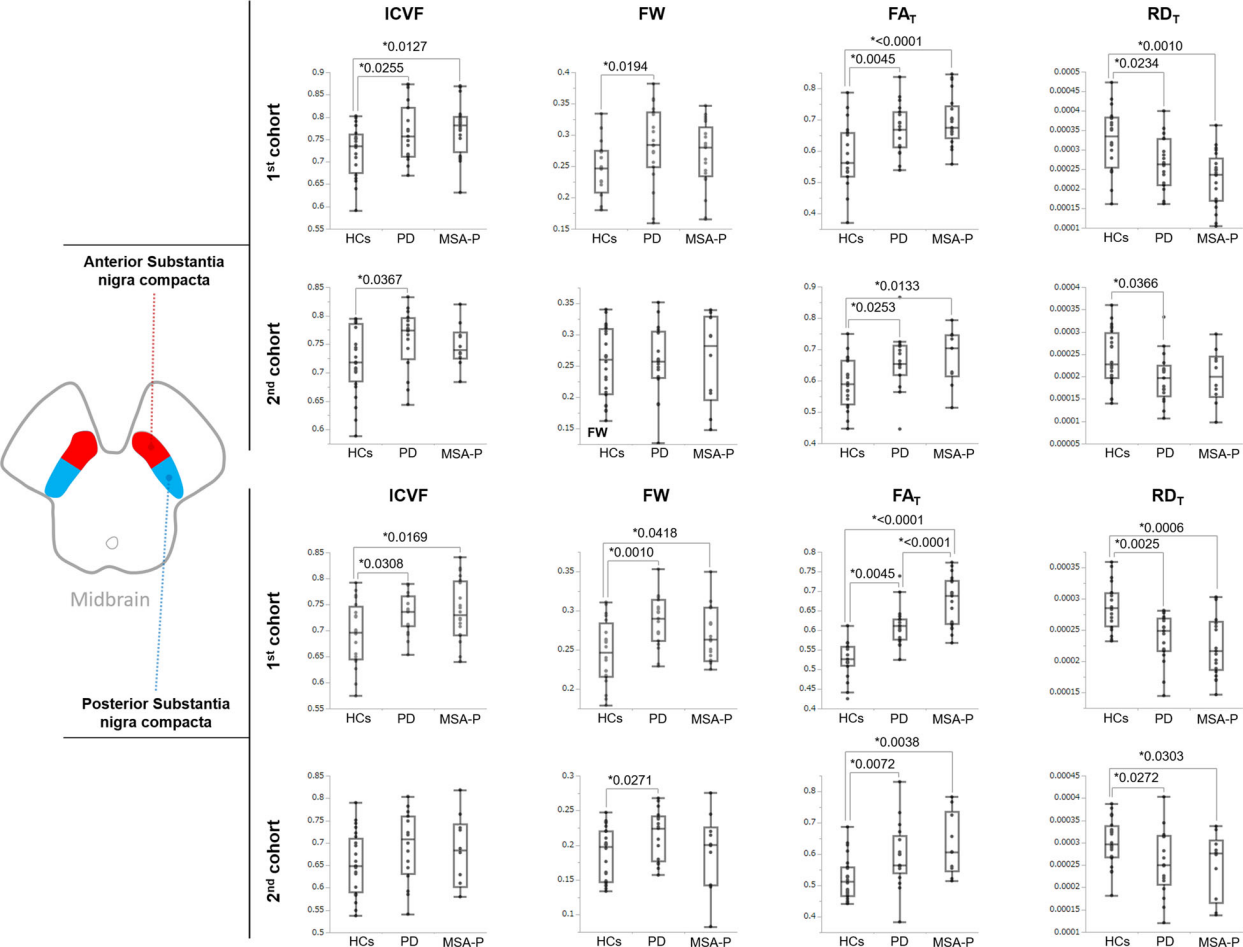
Results of the nigral ROI analysis. In the first cohort, both aSN and pSN showed significantly higher ICVF and FA_T_, and significantly lower RD_T_ in the MSA-P and PD patients compared with the HCs. The FW was significantly higher in aSN of PD patients compared with HCs, whereas the pSN showed significantly higher in both MSA-P and PD patients compared with HCs (Supplementary Table 4). Similar results were observed in the second cohort, especially in the FAT and RDT (Supplementary Table 4). In the boxplot, the center line represents the median, the box represents the third quartile, and the whiskers represent the maximum and minimum values. Abbreviations: FW free water, ICVF intracellular volume fraction, MVF myelin volume fraction, pSN posterior part of SNc, RD_T_ free water-corrected radial diffusivity, aSN anterior part of SNc, SNc substantia nigra pars compacta.

The substantia nigra pars compacta (SNc) was automatically applied to the automated anatomical labelling atlas 3 (AAL3)^20^, and the anterior (aSN) and posterior (pSN) regions were analyzed separately (Fig. 3). In the first cohort, both the aSN and pSN had significantly higher ICVF and FA_T_ in the MSA-P and PD patients compared with the HCs, and significantly lower RD_T_. The FW was significantly higher in the aSN of PD patients compared with HCs, whereas in the pSN it was significantly higher in both MSA-P and PD patients compared with HCs (Fig. 4, Supplementary Table 5). Similar results were observed in the second cohort, especially for FA_T_ and RD_T_ (Fig. 4, Supplementary Table 4).

### Correlation analysis

Spearman’s rank correlation analysis was used to investigate the correlations between significantly different MSA-P-specific regions from the ROI analysis and clinical features (Tables 2 and 3, first and second cohorts, respectively).

**Table 2:** Spearman rank correlations between significant regions in the ROI analysis and clinical features or MR indices in the first cohort.

		vs Clinical Features					vs Other Indices				
		DD	HY	UPDRS	UMSARS	LEDD	ICVF	FW	FA_T_	RD_T_	MVF
White matter											
MCP	ICVF	0.1	−0.27	−0.33	−0.11	−0.1	–	−0.54**	–	−0.95**	0.42**
	FW	0.07	0.39	0.3	0.08	−0.06	−0.54**	–	–	0.58**	−0.40**
	RD_T_	−0.1	0.3	0.37	0.18	0.04	−0.95**	0.58**	–	–	−0.40**
	MVF	0.19	−0.37	−0.47*	−0.2	0.01	0.42**	−0.40**	–	−0.40**	–
ICP	ICVF	0.13	−0.15	−0.36	−0.12	−0.18	–	−0.26**	–	–	0.38**
	FW	−0.12	0.54*	0.38	−0.1	−0.08	−0.26**	–	–	–	−0.49**
	MVF	0.12	−0.4	−0.28	−0.05	−0.17	0.38**	−0.49**	–	–	–
PCT	MVF	0.25	−0.45	−0.52*	−0.27	−0.04	–	–	–	–	–
CST	FW	−0.03	0.33	0.29	0.01	−0.01	–	–	–	–	–
SNc											
aSN	ICVF	−0.42	0.22	0.29	0.23	–0.094	–	0.20	0.46*	−0.51*	–
	FW	−0.042	0.0021	−0.30	−0.20	−0.038	0.20	–	−0.33	−0.067	–
	FA_T_	−0.086	0.39	0.48**	0.38	−0.23	0.46*	−0.33	–	−0.79**	–
	RD_T_	−0.055	−0.47*	−0.40	−0.36	0.37	−0.51*	−0.067	−0.79**	–	–
pSN	ICVF	0.054	0.39	0.053	0.066	−0.36	–	−0.057	0.65**	−0.83**	–
	FW	−0.051	0.25	−0.20	−0.18	−0.18	−0.057	–	−0.069	0.055	–
	FA_T_	0.13	0.61**	0.45*	0.36	−0.37	0.65**	−0.069	–	−0.91**	–
	RD_T_	−0.20	−0.52**	−0.30	−0.25	0.38	−0.83**	0.055	0.91**	–	–

*CST* corticospinal tract, *DD* disease duration, *FW* free water, *HY* Hoehn & Yahr stage, *ICVF* intracellular volume fraction, *ICP* inferior cerebellar peduncle, *MCP* middle cerebellar peduncle, *UPDRS* Movement Disorder Society-Unified Parkinson’s Disease Rating Scale part 3, *MVF* myelin volume fraction, *PCT* pontine crossing tract, *RD*_T_free water-corrected radial diffusivity, *SNc* substantia nigra pars compacta, *UMSARA* Unified Multiple System Atrophy Rating Scale part 2.

*FDR corrected *P* < 0.05; **FDR corrected *P* < 0.01.

**Table 3:** Spearman rank correlation results between regions showing significant differences in the ROI analysis and clinical features or MRI indices in the second cohort.

		vs Clinical Features					vs Other Indices				
		DD	HY	UPDRS	UMSARS	LEDD	ICVF	FW	FA_T_	RD_T_	MVF
White matter											
MCP	ICVF	0.39	−0.52**	−0.44	−0.29	−0.24	–	−0.21	–	−0.88**	–
	FW	−0.23	0.44	0.46	0.22	0.22	−0.21	–	–	0.19	–
	RD_T_	−0.48*	0.50**	0.52**	0.21	0.14	−0.88**	0.19	–	–	–
	MVF	–	–	–	–	–	–	–	–	–	–
ICP	ICVF	0.11	−0.41	−0.43	−0.43	−0.34	–	0.02	–	–	–
	FW	0.02	0.19	−0.02	0.13	0.12	0.02	–	–	–	–
	MVF	–	–	–	–	–	–	–	–	–	–
PCT	MVF	–	–	–	–	–	–	–	–	–	–
CST	FW	−0.2	−0.08	−0.04	−0.07	−0.22	–	–	–	–	–
SNc											
aSN	ICVF	0.14	0.27	0.34	0.31	0.36	–	−0.32	0.63**	−0.71*	–
	FW	0.076	−0.045	−0.20	−0.16	−0.28	−0.32	–	0.26	−0.25	–
	FA_T_	−0.12	0.27	0.018	−0.030	0.031	0.60	0.26	–	−0.84**	–
	RD_T_	−0.15	−0.31	−0.23	−0.14	−0.12	−0.71*	−0.25	−0.84**	–	–
pSN	ICVF	0.089	−0.13	−0.10	−0.43	0.18	–	0.36	0.84**	−0.84**	–
	FW	−0.13	−0.58	−0.41	−0.44	−0.48	0.36	–	0.042	−0.20	–
	FA_T_	0.32	0.31	0.29	0.32	0.44	0.84**	0.042	–	−0.87**	–
	RD_T_	−0.070	−0.045	0.055	0.049	−0.092	−0.84**	−0.20	−0.87**	–	–

*CST* corticospinal tract, *FW* free water, *ICVF* intracellular volume fraction, *ICP* inferior cerebellar peduncle, *MCP* middle cerebellar peduncle, *MDS-UPDRS* Movement Disorder Society-Unified Parkinson’s Disease Rating Scale, *MVF* myelin volume fraction, *PCT* pontine crossing tract, *RD*_T_ free water-corrected radial diffusivity, *ROI* region of interest, *SNc* substantia nigra pars compacta, *UMSARA* Unified Multiple System Atrophy Rating Scale.

*FDR corrected *P* < 0.05; **FDR corrected *P* < 0.01.

In the first cohort white matter analyses, the FW results had a significant (FDR-corrected *P* < 0.05) positive correlation with HY stage in the ICP, whereas MDS-UPDRS part 3 scores were negatively correlated with MVF in the PCT and MCP (Table 2; vs. clinical features). For the MCP and ICP, which showed MSA-P-specific changes, there were correlations between all parameters. In the MCP, there were significant (FDR-corrected *P* < 0.05) positive correlations between FW and RD_T_, and between MVF and ICVF, whereas there were negative correlations between the FW and ICVF, RD_T_ and ICVF, MVF and FW, and MVF and RD_T_. In the ICP, there was a positive correlation between the MVF and ICVF, and there were negative correlations between the FW and ICVF and between the MVF and FW (Table 2; vs. other indices). In the second cohort, there were significant (FDR-corrected *P* < 0.05) negative correlations between the disease duration and RD_T_ of the MCP, and between the HY stage and ICVF of the MCP. There were also significant positive correlations between HY stage and RD_T_ of the MCP, and between MDS-UPDRS part 3 scores and RD_T_ of the MCP (Table 3; vs. clinical features). Comparisons between the other indices, ICVF, and RD_T_ revealed significant (FDR-corrected *P* < 0.05) negative correlations in the MCP (Table 3; vs. another model).

In the first cohort, the SNc had significant (FDR-corrected *P* < 0.05) positive correlations between the MDS-UPDRS part 3 scores and FA_T_ in the aSN, and HY stage and MDS-UPDRS part 3 scores were positively correlated with FA_T_ in the pSN. Moreover, there were significant negative correlations between MDS-UPDRS part 3 scores and HY stage and RD_T_ in the aSN, and HY stage and RD_T_ in the pSN (Table 2; vs. clinical features). In both the aSN and pSN, there were significant (FDR-corrected *P* < 0.05) positive correlations between the ICVF and FA_T_, and significant negative correlations between ICVF and RD_T_, and FA_T_ and RD_T_ (Table 2; vs. other indices). In the second cohort, there were no clear correlations between any SNc parameters and clinical features (Table 3; vs. clinical features). However, the correlation results between SNc parameters and other indices were consistent with those in the first cohort (Table 3; vs. other indices).

### Nominal logistic regression analysis and receiver operating characteristics (ROC) curves

**Fig. 5:**
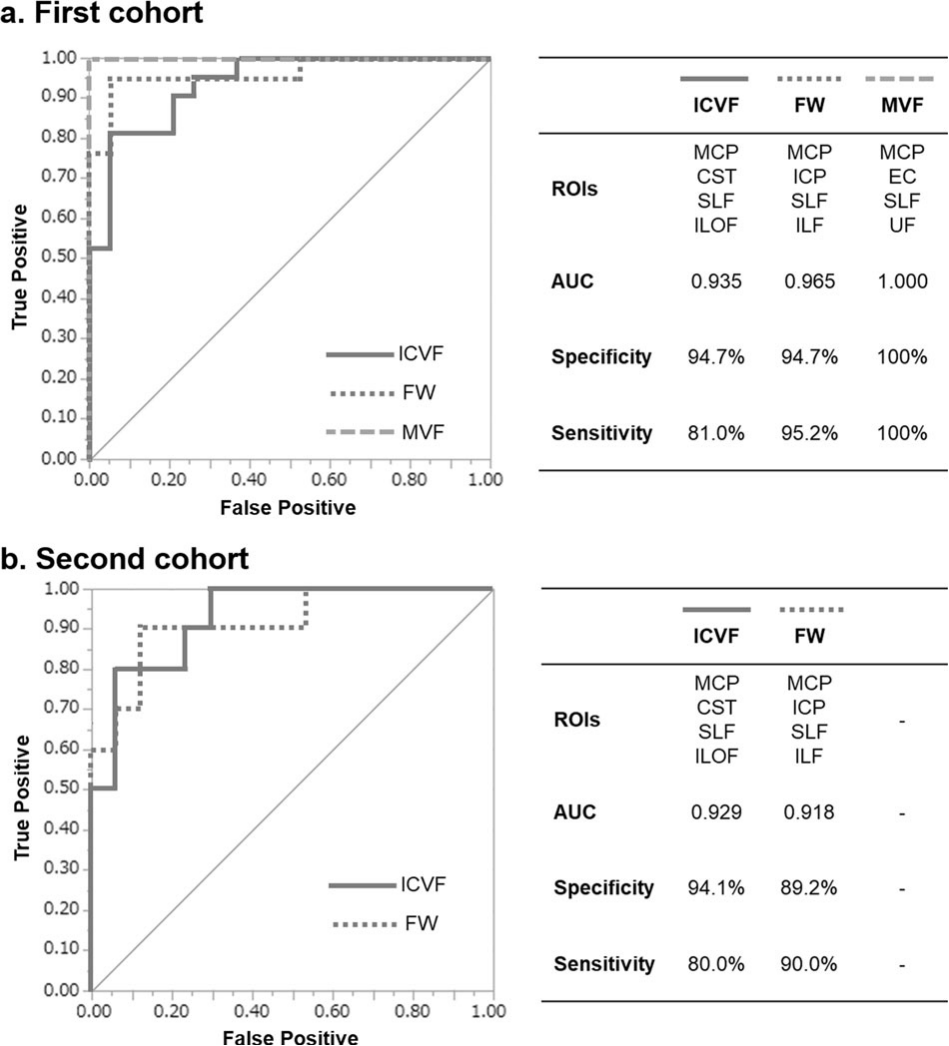
ROC curve for distinguishing MSA-P from PD. **a** In the first cohort, the AUC for ICVF was 0.935 (specificity 94.7%, sensitivity 81.0%) using the four regions of MCP, CST, SLF, and ILOF. The AUC for FW was 0.965 (specificity 94.7%, sensitivity 95.2%) using the MCP, ICP, SLF, and ILF. The AUC for MVF was 1.000 (Specificity 100%, Sensitivity 100%) when using MCP, EC, SLF, and UF. **b** Similar AUCs for ICVF and FW were also found for the second cohort (Supplementary Table 5). CST corticospinal tract, EC external capsule, FW free water, ICP inferior cerebellar peduncle, ICVF intracellular volume fraction, ILF inferior longitudinal fasciculus, ILOF inferior fronto-occipital fasciculus, MCP middle cerebellar peduncle, MVF myelin volume fraction, SLF superior longitudinal fasciculus.

Stepwise forward logistic regression analysis revealed that, from the 30 ROIs in ICVF, FW, and MVF in which multiple changes were noted in the white matter (Supplementary Table 5), four tracts might be useful for differentiating MSA-P from PD. ROC analysis was performed to confirm the diagnostic benefits for MSA-P. In the first cohort, the area under the curve (AUC) for ICVF was 0.935 (specificity 94.7%, sensitivity 81.0%) when using the four regions of the MCP, CST, superior longitudinal fasciculus (SLF), and inferior fronto-occipital fasciculus (IFOF). For FW, the AUC was 0.965 (specificity 94.7%, sensitivity 95.2%) when using the MCP, ICP, SLF, and inferior longitudinal fasciculus (ILF). For MVF, the AUC was 1.000 (specificity 100%, sensitivity 100%) when using the MCP, EC, SLF, and uncinate fasciculus (UF) (Fig. 5a, Supplementary Table 5). In the second cohort, we examined whether the tracts selected in the first cohort were able to continue to differentiate MSA-P from PD. Both the ICVF and FW results had AUCs over 0.9 using the same regions as used for the first cohort (Fig. 5b, Supplementary Table 5).

## Discussion

In the present study, we used advanced diffusion MRI (NODDI, FWE-DTI) and myelin-sensitive imaging (MT-sat) to examine the white matter and SNc microstructure in HCs, PD patients, and MSA-P patients. These advanced diffusion MRI and myelin-sensitive imaging techniques were able to capture alterations specific to MSA-P patients, which involve white matter degeneration and myelin changes related to oligodendrogliopathy. MRI has been used to identify the characteristic pathological changes of MSA in the pons, cerebellum, striatum, and substantia nigra. MSA can be diagnosed from conventional MRI sequences if characteristic findings are depicted, such as the pontine cross, hyperintense MCP, and putaminal slit sign^1^. However, not all MSA-P patients have these specific MRI findings (Table 1); therefore, various studies using MRI, including diffusion MRI, are required to improve the diagnosis of MSA-P at earlier stages and to identify other characteristic changes.

Our TBSS analysis of DTI identified diffuse white matter alterations (Fig. 1a). A decrease in FA and an increase in MD suggests neurodegeneration^21^, whereas an increase in RD suggests demyelination^22^. An increased MD and RD compared with HCs^23^, and a decreased FA and increased MD compared with PD, especially in the MCP^24,25^, have been previously reported in MSA-P patients, and we found similar results in the present study. However, in the current study, patients with MSA-P had no significant differences in FA compared with PD patients. This is probably because FA overestimates neurodegeneration in areas that are rich in crossing fibers and FW^14,26,27^. However, we revealed that FA_T_ was different between MSA-P and PD patients in a small area. Thus, as previously reported^14^, to elucidate the white matter microstructural alterations that occur in MSA, FW signals should be eliminated from the usual DTI^23–25,27^.

We analyzed white matter alterations in MSA-P patients using multishell bi-tensor NODDI and FWE-DTI. TBSS and ROI analysis of the white matter showed MSA-P-specific alterations, with decreased ICVF and increased FW in the MCP, ICP, and CST (Figs. 1d, 2). Decreased ICVF has been reported to reflect a sparsity of neurites in the white matter^7,8^, and increased FW can be caused by abnormal extracellular FW space, which is associated with neuroinflammation, axon degeneration, and demyelination^11^. Considering the pathology of MSA-P as an oligodendrogliopathy caused by α-synuclein aggregation, the accumulation of GCIs might induce neuroinflammation^28^, leading to demyelination and a loss of neurites. Thus, the decreased ICVF and increased FW that was captured by diffusion MRI in the present study might reflect the pathological condition of MSA. A previous report revealed that [11 C](R)-PK11195 positron emission tomography (PET) might be useful for reflecting neuroinflammation caused by microglial activation in MSA^29^. In the future, comparisons between FW imaging and [11 C](R)-PK11195 PET might help to clarify the mechanisms of in vivo neuroinflammation in MSA.

Previous studies have reported increased FW in the substantia nigra, striatum, globus pallidus, red nucleus, thalamus, pedunculopontine nucleus, MCP, superior cerebellar peduncle, vermis, cerebellar lobule, and corpus callosum of MSA patients, as well as increased FA_T_ in the striatum^16,17^. Mitchell et al. also reported NODDI and FW alterations in the basal ganglia, thalamus, cerebellum, and brainstem of MSA patients compared with HCs, PD patients, MSA-P patients, and progressive supranuclear palsy patients^18^. The study design of Mitchell et al. was similar to ours, although they used single-shell diffusion-weighted imaging (DWI) data, whereas we used multishell DWI. Multishell DWI might provide more detailed information on brain microstructures and alterations in extracellular FW^30^. In addition, no FW studies have extensively focused on white matter microstructure in MSA^31–34^. Although MT-sat (a myelin-sensitive technique) has been demonstrated to have good sensitivity for the detection of demyelinating white matter lesions^35^, only a few studies have used myelin-sensitive imaging to examine the white matter of MSA patients. In one study, differences in the magnetization transfer ratio were reported in the precentral gyrus only^36^, whereas another study reported no significant white matter alterations^37^. However, MT-sat improves the magnetization transfer ratio and can better delineate demyelinating lesions, consistent with clinical symptoms^38^. In the present study, specific changes in MVF were observed in MSA-P patients compared with HCs or PD patients (Figs. 1d, 2), whereas there were no differences between the HCs and PD patients. Thus, MVF was able to clearly distinguish between the PD and MSA patients. Consistent with pathological findings, the most prominent alterations in MVF occurred in the brainstem and cerebellum, and were indicative of severe demyelination. Similar to MVF, RD, which can also be used to detect demyelination^22^, showed widespread changes in the white matter, cerebellum, and brainstem in the present study. However, widespread RD changes were also observed in PD patients who had no pathological evidence of demyelination. We therefore speculate that RD might be affected by regions with many crossing fibers, as well as by disease-induced white matter microstructural alterations^39^. Thus, MVF may be more sensitive than RD for the detection of demyelination in MSA patients.

The characteristic conventional MRI findings of MSA-P (shown in Table 1) provide a very good diagnostic basis. Because the present research focused on identifying microstructural alterations of MSA-P patients, we selected cases with sufficient diagnostic evidence of MSA-P, and also examined diagnostic accuracy. Several parameters, including ICVF from NODDI, FW from FWE-DTI, and MVF from MT-sat were useful for discriminating between the MSA-P and PD (Fig. 5). In particular, MVF in the MCP, EC, SLF, and UF reached 100% sensitivity and specificity for this discrimination, while ICVF in the MCP, CST, SLF, and IFOF reached a specificity of about 95%, and FW in the MCP, ICP, SLF, and ILF reached a sensitivity and specificity of about 95%. The sensitivity and specificity of ICVF and FW were confirmed in the second cohort, with the AUCs exceeding 0.9 (Fig. 5a, b). These results suggest that capturing white matter abnormalities may be important for understanding the pathophysiology of MSA-P.

ROI analysis revealed alterations of ICVF, MVF, FW, and RD_T_ in the MCP, ICP, PCT, and CST of MSA-P patients compared with HCs and PD patients (Fig. 2). Correlation analyses performed on the two cohorts indicated that MDS-UPDRS part 3 scores and HY stage were associated with MVF and RD_T_ of the MCP (Tables 2 and 3). These data indicate that there are associations between the state of white matter microstructures and disease progression, with the deterioration of motor symptoms. These effects on motor function may be associated with cerebellar dysfunction; in general, cerebellar ataxia is closely correlated with impaired motor function^40^.

The parameters of ICVF, FW, RD_T_, and MVF in the MCP, and the measures of ICVF, FW, and MVF in the ICP, were correlated with one another (Table 2). In the second cohort, although there were fewer participants than in the first cohort, a negative correlation (*r*_*s*_ = −0.88) between the RD_T_ and ICVF was also confirmed (Table 3). Increased RD_T_ and decreased ICVF may reflect demyelination and a decreased density of neurites in the white matter^41^. Considering the MT-sat findings, alterations in these parameters may reflect oligodendrogliopathy in MSA. On the basis of the ROI analysis of the first cohort, MSA-P-specific changes in FW, MVF, and ICVF can be divided into three categories: elevated FW and decreased MVF and ICVF, as found in the MCP and ICP; decreased MVF only, as found in the PCT; and elevated FW only, as found in the CST. These findings suggest that the MCP and ICP may be more vulnerable than other regions to white matter degeneration caused by neurite loss and demyelination. Although the MCP sign in conventional MRI was only observed in 4.8% of patients in the present study (Table 1), the microstructure was highly impaired. PCT degeneration can be seen as a pontine cross sign on conventional MRI. MVF revealed that demyelination might occur in the PCT, although it was only identified in 9.5% of patients in the present study. In the CST, FW was significantly higher and MVF was significantly lower compared with the HCs, but MVF was not significantly different from that of PD patients. These changes in the PCT and CST were different between the two cohorts, but the tendency of the mean value was the same (Fig. 2, Supplementary Table 4), and it was considered that the difference in tract impairment affected the results. Considering the MCP/ICP changes and MSA pathology, it is possible that changes in FW and ICVF may also be observed in the PCT and CST as the disease progresses. The different distributions of ICVF, FW, and MVF in regions where pathological changes have been reported in MSA indicate that a combination of these parameters might help us to understand which neural structures are impaired.

In the ROI analysis of the SNc, both the cohorts consistently had increased FA_T_ (in both anterior and posterior parts) and decreased RD_T_ (in the posterior part only) in patients with PD and MSA-P compared with HCs. These findings might indicate the degeneration of dopaminergic neurons in the SNc in both diseases. Our results are also in line with those of previous studies showing a stronger neurodegenerative process in the posterior part of the SNc^16–18^.

Neurodegenerative diseases such as PD or MSA have generally been associated with decreased FA in the substantia nigra^42–44^. However, these findings have not always been consistent. No differences between groups or increased FA have been reported in the substantia nigra of patients with PD compared with HCs^45,46^. The conflicting findings may be partly caused by discrepancies in sample characteristics (e.g., disease duration or symptom severity) and acquisition schemes. Moreover, the correct identification of each ROI is critical to study results. Previous studies in PD and MSA-P patients have used different ROI settings, such as manual or automated identification of the whole substantia nigra (including the substantia nigra pars reticulata) or SNc^47^. Considering that dopaminergic neuronal loss in the SNc is considered a common pathological feature of PD and MSA-P^2^, measurement of the whole substantia nigra might have reduced the diagnostic accuracy. Importantly, DTI-derived measures lack specificity. For example, decreased FA may be attributed to neuronal degeneration or neuroinflammation^48^. In the current study, we evaluated the SNc in two cohorts (with different diffusion MRI acquisition parameters) of patients with PD and MSA-P using the AAL3 atlas (Fig. 3)^20^. A fully automatic method was applied to ensure the accuracy of SNc segmentation in each subject. Furthermore, we performed FWE-DTI to eliminated the influence of extracellular FW from DTI-derived measures, allowing more specific measurements of tissue pathologies. Both the cohorts of PD and MSA-P patients showed consistent results (e.g., increased FA_T_ or decreased RD_T_) in the SNc compared with HCs. Therefore, we can reasonably claim the robustness of our results.

Increases in iron concentrations in the SNc, as occurs in many neurodegenerative diseases, may lead to dopaminergic cell death^49^. It is well recognized that nigral iron accumulation contributes to changes in DTI-derived measures, including FA elevation. A longitudinal study over 12 years (with 6-year intervals) in the deep gray nuclei of PD patients demonstrated increased FA at the 6-year follow-up and decreased FA at the 12-year follow-up, which suggests iron deposition and neuronal loss, respectively^50^. Furthermore, significantly higher FA_T_ was observed in the pSN of patients with MSA-P (in the first cohort) compared with PD patients in the present study. Pathological studies have demonstrated that iron levels in the substantia nigra are higher in patients with MSA-P than in those with PD^51–53^, suggesting that patients with MSA-P have more severe alterations in the SNc compared with PD patients. Indeed, significantly higher HY stages and MDS-UPDRS part 3 scores were observed in MSA-P patients compared with PD patients (Table 1). In addition, decreased RD_T_ was consistently demonstrated in the pSN in both the cohorts of PD and MSA-P patients compared with HCs, and RD has been reported to decrease as local iron concentrations increase^54^.

Different pathological processes, such as gliosis, where the infiltration of gliotic cells increases tissue anisotropy, may also be responsible for increased FA in the SNc^55^. Furthermore, a functional connectivity or pathological study has demonstrated widespread connectivity between the SNc and other brain regions such as the substantia nigra pars reticulata, subthalamic nucleus, limbic system, and frontal cortex^56^. Hence, the SNc is thought to contain a large number of different fibers^57^. Although SNc dopaminergic nerves are degenerated in PD and MSA-P, other nondopaminergic fibers are relatively conserved; therefore, a selective loss of specific fiber directions in the crossing-fiber areas may also increase FA^58^. Future studies, including neuromelanin-sensitive imaging^59^ and susceptibility-weighted imaging^60^, are warranted to set appropriate ROIs in the SNc and explore iron accumulation with higher specificity.

In the present study, significantly higher ICVF was also demonstrated in the aSN and pSN in patients with MSA-P compared with HCs (in the first cohort only). Similar to FA_T_ and RD_T_, the changes in ICVF might also be attributed to iron accumulation and/or gliosis. This concept is reinforced by the finding that ICVF was significantly correlated with FA_T_ and RD_T_ (Tables 2 and 3). However, further histopathological studies are needed to clarify the underlying mechanisms of increased ICVF.

Considering the neurodegeneration of SNc that occurs in synucleinopathies, our results might reflect the precise histological state of the SNc in vivo^2,61^. Moreover, we revealed that HY stage was significantly correlated with RD_T_ of the aSN, and with FA_T_ and RD_T_ of the pSN, and that MDS-UPDRS part 3 scores were significantly correlated with FA_T_ of the aSN and pSN. These results suggest that alterations in FA_T_ and RD_T_ in the substantia nigra might be a useful biomarker for evaluating motor dysfunction in synucleinopathies.

The present study had several limitations. First, the diagnoses of PD and MSA-P were carefully determined by neurologists and radiologists based on diagnostic criteria and neuroimaging findings, but were not pathologically confirmed. Second, this was a cross-sectional study, and a longitudinal study is needed to elucidate the pathogenesis of MSA and evaluate disease progression. Third, it should be noted that the presence of FW producing neuroinflammation has not been fully validated by pathological or PET studies. Finally, although the MRI data were acquired using a sufficiently uniform procedure, the effects of head angles at the time of imaging need to be taken into consideration. However, in the current study, there were no differences between the groups in head angle (as calculated by the method shown in Supplementary Figure. 1).

In conclusion, the present study used two different MRI protocols and cohorts and revealed that multi-shell bi-tensor NODDI, FWE-DTI, and MT-sat can detect white matter and SNc microstructural alterations in MSA-P patients compared with PD patients and HCs. Our data indicate that a combination of these imaging parameters has the potential to identify differences in the degeneration of specific regions. From the results of this study, white matter alterations of ICVF, FW, and MVF, and SNc alterations of ICVF, FA_T_, and RD_T_ may be useful for evaluating neurodegeneration. Our results may help to understand the alterations in brain microstructure that occur in MSA-P. Further studies combined with histological analysis to elucidate the pathology of neurodegeneration in MSA are needed.

## Methods

### Participants

This study was conducted in compliance with the Declaration of Helsinki (1964, latest update in 2013) and received ethical approval from Juntendo University (14-011). Informed written consent was obtained from all participants. Clinical data were carefully evaluated by three movement disorder specialists (T.O., T.H., and H.T.A.), and cases with apparent cognitive impairment based on the Mini-Mental State Examination^62^ or minimal vascular lesions were excluded from the study. Patients who met the criteria for the diagnosis of probable MSA-P^1^, age- and sex-matched patients with clinically established PD^63^ who met the Movement Disorder Society Clinical Diagnosis Criteria for Parkinson’s Disease, and age- and sex-matched HCs were enrolled in this study. All MSA-P patients were followed for more than 3 years and the presence of other neurodegenerative diseases was ruled out.

The analyses performed on the first cohort were followed by a second set of analyses on a second cohort to corroborate the findings. The first cohort was made up of 21 patients with MSA-P (62.5 ± 11.7 years; eight men), 19 patients with PD (63.1 ± 8.1 years; seven men), and 20 HCs (62.8 ± 4.7 years; eight men). The second cohort was made up of 10 patients with MSA-P (65.3 ± 9.5 years; seven men), 17 patients with PD (63.2 ± 10.2 years; 10 men), and 24 HCs (65.8 ± 6.5 years; 13 men). In the first cohort, data were collected between January 2017 and December 2018, and in the second cohort, data were collected between January 2019 and November 2020.

Clinical information from the MSA-P and PD patients was examined, including disease duration, HY stage^64^, the Japanese translation version of MDS-UPDRS part 3^65^, the UMSARS part 2 (only for MSA-P patients)^66^, the LEDD (calculated from the conversion rate of anti-parkinsonian medications)^67^, and a single-question screen for RBD^68^. Structural MRI findings on T1-WI (repetition time [TR] = 2300 ms, echo time [TE] = 2.32 ms, inversion time [TI] = 900 ms), T2-WI (TR = 4500 ms, TE = 88 ms), T2*-WI (TR = 500 ms, TE = 12 ms), and fluid-attenuated inversion recovery imaging (TR = 10,000 ms, TE = 114 ms, TI = 2640 ms) were also obtained, including the pontine cross sign, vertical hyperintensity line in the pons, cerebellar atrophy, the MCP-sign, and the putaminal slit.

### Acquisition of magnetic resonance imaging data

All study participants were scanned on a 3T-MRI scanner (MAGNETOM Prisma, Siemens Healthcare, Erlangen, Germany) using a 64-channel head coil. Multi-shell DWI was performed using a spin-echo echo-planar imaging sequence, which included two b values of 1000 and 2000 s/mm^2^ in the first cohort, and two *b* values of 700 and 2000 s/mm^2^ in the second cohort. The DWI data were obtained using an anterior–posterior phase-encoding direction along 64 isotropic diffusion gradients for each shell. Acquisition of each DWI dataset was completed with a *b* = 0 image with no diffusion gradients. To correct for magnetic susceptibility induced distortions related to the echo-planar imaging acquisitions, standard and reverse phase-encoded blipped images with no diffusion weighting (blip up and blip down) were obtained^27,69^. The sequence parameters used for the first cohort were TR = 3300 ms, TE = 70 ms, field of view = 229 × 229 mm, matrix size = 130 × 130, resolution = 1.8 × 1.8 mm, slice thickness = 1.6 mm, and acquisition time = 07.29 min. The sequence parameters used for the second cohort were TR = 3600 ms, TE = 79 ms, field of view = 204 × 204 mm, matrix size = 120 × 120, resolution = 1.7 × 1.7 mm, slice thickness = 1.7 mm, and acquisition time = 7.04 min (Supplementary Table 6).

To calculate the MT-sat index (the sequence for which was only acquired in the first cohort), predominant T1-WI, proton density-WI, and MT-WI were acquired using three-dimensional multi-echo fast low-angle shot sequences. The settings for the MT-sat sequences were as follows: for MT-off and MT-on scanning, TR = 24 ms, TE = 2.53 ms, flip angle = 5°; for T1-WI, TR = 10 ms, TE = 2.53 ms, flip angle = 13°, with parallel imaging using GeneRalized Autocalibrating Partially Parallel Acquisitions with a factor of 2 in the phase-encoding direction, 7/8 partial Fourier acquisition in the partition direction, bandwidth = 260 Hz/pixel, field of view = 224 × 224 mm, matrix = 128 × 128, slice thickness = 1.8 mm, and acquisition time = 6 min 25 s.

### MRI pre-processing

Head angle was calculated to rule out the effects of head position on the acquired data. The actual head angle was measured by correcting the oblique angle at imaging from the DICOM header information and calculating the angle on the image from the rotation matrix when standardizing the T1-WI images (Supplementary Figure 1). The calculated data confirmed that there were no significant differences (*P* > 0.05) between the disease groups.

The EDDY and TOPUP toolboxes were used to correct susceptibility-induced geometric distortions, eddy current distortions, and inter-volume subject motion in the DWI datasets^27^. All DWI datasets were then visually checked in the axial, sagittal, and coronal views, and were confirmed to be free from severe artifacts, such as gross geometric distortion, signal dropout, and bulk motion. Multishell DWI data were used to generate NODDI and FWE-DTI maps^70^. The NODDI model^7^ was applied to the MRI results using the NODDI Matlab Toolbox5 (http://www.nitrc.org/projects/noddi_toolbox). The ICVF, ODI, and ISOVF maps were calculated using AMICO (Accelerated Microstructure Imaging via Convex Optimization). FWE-DTI data were processed using a regularized bi-tensor model in MATLAB (MathWorks, Natick, MA, USA)^14^, and FA_T_, MD_T_, RD_T_, AD_T_, and FW maps were calculated. ISOVF and FW, which can be obtained from NODDI and FWE-DTI, respectively, are both indicators of extracellular FW content in the brain.

The DTI measures were obtained using an ordinary least square method applied to the DWI with *b* = 0 and 1000 s/mm^2^. FA, MD, AD, and RD maps were calculated using the DTIFIT tool implemented in FSL (FMRIB Software Library 5.0.9; Oxford Centre for Functional MRI of the Brain, UK; www.fmrib.ox.ac.uk/fsl), which is based on standard formulae^70^. MT-sat was calculated using an in-house MATLAB script based on previously described theory^71^. MVF maps (calculated for the first cohort only) were obtained using an MT-sat correction factor of 0.1.

### Tract-based spatial statistics analysis

We evaluated participants’ white matter alterations using a TBSS skeleton projection step^72^. First, nonlinear registration of FA images of all subjects was mapped onto MNI (152) space and interpolation to a 1 × 1 × 1 mm resolution was performed using the FMRIB nonlinear registration tool. Second, the transformed FA images were averaged to create a mean FA image. Third, the mean FA image was thinned to create a mean FA skeleton, which represented the centers of all tracts common to the groups. The threshold of the mean FA skeleton was set to >0.20 to include the major white matter pathways but exclude peripheral tracts and gray matter. The aligned FA map of each subject was then projected onto the skeleton. Finally, the DTI (MD, AD, and RD), NODDI (ICVF, ODI, and ISOVF), FWE-DTI (FA_T_, MD_T_, RD_T_, AD_T_, and FW), and MT-sat (MVF) maps were projected onto the mean FA skeleton after being registered to MNI space using the warping fields of each subject.

### Region-of-interest analysis

White matter was further evaluated using automated ROI analysis. Maps showing significant clusters on TBSS analyses were localized using the John Hopkins University (JHU) white matter tractography atlas and the International Consortium of Brain Mapping (ICBM)-DTI-81 white matter atlas^73^. The 30 ROIs were as follows: the MCP, ICP, superior cerebellar peduncle, PCT, medial lemniscus, CST, cerebral peduncle, genu/body/splenium of corpus callosum, anterior/posterior limb of internal capsule, retrolenticular part of internal capsule, anterior/superior/posterior corona radiata, forceps, anterior/posterior thalamic radiation, IFOF, ILF, SLF, superior fronto-occipital fasciculus, EC, cingulum cingulate gyrus, cingulum hippocampus, fornix/stria-terminalis, UF, and tapetum.

The SNc ROI was automatically created using the AAL3^20^. The ROIs of the aSN and pSN were manually divided in the middle of each section of the SNc (Fig. 3). In the SNc ROI analysis, the signal-to-noise ratio can decrease because of the surrounding iron-rich region, resulting in fitting errors. To rule out the possibility of this artifact, voxels with an ICVF of 0.99 or higher were excluded from the analysis.

We also considered the possibility that the AAL3, automatically adapted to standard space, might overestimate the surrounding structures, such as the white matter or cerebrospinal fluid. A square ROI that fit in the SNc was therefore created manually using ITK-SNAP^74^ with reference to AAL3, and the same analysis was performed (Supplementary Method 1).

### Statistical analysis

All analyses were performed using statistical software (JMP v14; SAS Inc., Cary, NC, USA; or the FSL package for the general linear model analysis). Significance was defined as *P* < 0.05, corrected for multiple comparisons. The background demographics of participants were analyzed using Wilcoxon analysis or Kruskal−Wallis analysis for continuous variables, such as age, disease duration, HY stage, MDS-UPDRS part 3 scores, and LEDD, and the Pearson’s chi-squared test was used for nominal variables.

Thirty white matter ROIs were selected based on the JHU and ICBM atlases, and these were used to compare the overall white matter differences between MSA-P patients, HCs, and PD patients. In the 30 ROI analyses, the averaged values of the left and right part of each region were used. For each ROI in the white matter and SNc, Kruskal−Wallis analysis was performed for group comparisons (e.g., HCs vs. MSA-P patients vs. PD patients), and the results were corrected for multiple tests using the Benjamini–Hochberg FDR method^75^. *Post-hoc* analyses (HCs vs. MSA-P patients, HCs vs. PD patients, and MSA-P patients vs. PD patients) of NODDI, FWE-DTI, and MT-sat measures were calculated using Steel–Dwass analysis.

Spearman’s rank correlation was used to examine correlations between MRI parameters within regions that showed significant differences in the ROI analyses of the white matter and SNc of MSA-P patients, and patient characteristics including disease duration, HY stage, MDS-UPDRS part 3 scores, UMSARS scores, and LEDD. Correlations between the parameters of the different MRI models were also evaluated using Spearman’s rank correlation for regions that showed MSA-P-specific differences.

We used stepwise logistic regression analysis to select the four tracts that were needed to distinguish MSA-P patients from PD patients from the 30 white matter ROIs. The forward method was used, and the inclusion *P* values were set to 0.05. Furthermore, a ROC analysis was performed using the obtained four tracts, and the AUC, specificity, and sensitivity were calculated.

### Reporting Summary

Further information on research design is available in the Nature Research Reporting Summary linked to this article.

## Supplementary information

Supplementary Information

Reporting Summary

## Data Availability

The data supporting the findings of this study are available from the corresponding author upon reasonable request.

## Code availability

The tools used for MRI pre-processing in this paper are available from the following; EDDY, TOPUP and DTIFIT toolboxes (FSL, FMRIB Software Library; http://www.fmrib.ox.ac.uk/fsl), Matlab Toolbox5 (http://www.nitrc.org/projects/noddi_toolbox), AMICO (Accelerated Microstructure Imaging via Convex Optimization), and Matlab (MathWorks, Natick, MA, USA). The FMRIB nonlinear registration tool used for TBSS is available from FSL (http://www.fmrib.ox.ac.uk/fsl). The JHU white matter tractography atlas (Johns Hopkins Medical Institute Laboratory of Brain Anatomical MRI; http://www.https://cmrm.med.jhmi.edu/) and the ICBM-DTI-81 white matter atlas (UCLA Brain Mapping Center; http://www.http://www.bmap.ucla.edu/portfolio/atlases/ICBM_DTI-81_Atlas/) are used for ROI analysis. All other statistical analyses were performed using SAS JMP version 14 (SAS Inc., Cary, NC, USA).
