# Supplementary material for: White matter and nigral alterations in multiple system atrophy-parkinsonian type
Source: NPJ Parkinsons Dis. 2021 Oct 29;7:96. doi: 10.1038/s41531-021-00236-0 (PMC8556415; doi:10.1038/s41531-021-00236-0)
Supplement: Supplementary file 1 — Supplementary Information [file 41531_2021_236_MOESM1_ESM.pdf]

Supplementary Table 1. Outline of DTI, NODDI, FWE-DTI, and MT-sat parameters

**Diffusion tensor imaging (DTI) (Schocke MFH, et al. Neurology 2002; Schocke MFH, et al Neuroimage 2004)**	
Fractional anisotropy (FA)	Overall directionality of water diffusion within brain tissue
Mean diffusivity (MD)	Magnitude of isotropic diffusion within brain tissue
Axial diffusivity (AD)	Magnitude of isotropic diffusion within brain tissue along the direction of maximal diffusion
Radial diffusivity (RD)	Magnitude of isotropic diffusion within brain tissue perpendicular to direction of maximal diffusion
**Neurite orientation dispersion and density imaging (NODDI) (Zhang H, Neuroimage 2012)**	
Intracellular volume fraction (ICVF)	Density of neurites (axons and dendrites) based on intracellular diffusion
Orientation dispersion index (ODI)	Dispersion of neurites (axons and dendrites) in the intracellular compartment
Isotropic volume fraction (ISOVF)	Volume fraction of isotropic diffusion: ex. cerebrospinal fluid
**Free-water eliminated DTI (FWE-DTI) (Pasternak O, et al. Magn Reson Med 2009)**	
Free water (FW)	Fractional volume of free-water: extracellular free water, captures a non-flowing, non-diffusing water molecule, and might reflects on the neuroinflammation
Free water-corrected FA (FA_T),	Similar as DTI
Free water-corrected MD (MD_T),	Similar as DTI
Free water-corrected AD (AD_T),	Similar as DTI
Free water-corrected RD (RD_T),	Similar as DTI
**Magnetization transfer-saturation (MT-sat) imaging (Duval T, et al. Funct neurol 2016)**	
Myelin volume fraction (MVF)	Myelin sensitive imaging; decreasing value indicates demyelination

Supplementary Table 2. Results of tract-based spatial statistics (TBSS) analysis for multiple system atrophy-parkinsonian type (MSA-P) patients, Parkinson's disease (PD) patients, and healthy controls (HCs) in the first cohort

**Modality**	**Contrast**	**Cluster size**	**Anatomical region**	**Peak *T*-value**	**Peak MNI coordinates (X, Y, Z)**
**DTI**					
FA	HCs > MSA-P	48890	Bilateral ATR, CST, cingulum hippocampus, IFOF, ILF, SLF, UF, SLF temporal part, RIC, ACR, SCR, PCR, PTR, sagittal stratum, external capsule, fornix stria terminalis; left CC; right ALIC, PLIC, SFOF; forceps minor and major, middle cerebellar, tapetum peduncle, fornix, genu, body and splenium of CC.	5.09	(122, 118, 81)
	HCs > PD	45703	Bilateral ATR, CST, cingulum hippocampus, IFOF, ILF, SLF, UF, SLF temporal part, RIC, ACR, SCR, PCR, PTR, sagittal stratum, external capsule, fornix stria terminalis; right ALIC, PLIC, SFOF, tapetum; forceps major and minor, middle cerebellar peduncle, fornix, genu, body and splenium of CC.	7.97	(60, 148, 108)
MD	HCs < MSA-P	59307	Bilateral ATR, CST,IFOF, ILF, SLF, UF, SLF temporal part, medial lemniscus, ICP, SCP, cerebral peduncle, ALIC, PLIC, RIC, ACR, SCR, PCR, PTR, sagittal stratum, external capsule, fornix stria terminalis, SLF, SFOF, UF; left CCG; right tapetum; forceps major and minor, middle cerebellar peduncle, pontine crossing tract, genu, body and splenium of CC, fornix.	8.35	(121, 118 ,82)
	HCs < PD	4074	Bilateral ATR, IFOF, ACR, SCR; left CST; right AIC, PIC, external capsule, SFOF; forceps minor, genu and body of CC.	4.71	(58, 160, 74)
	PD < MSA-P	25032	Bilateral ATR, CST, IFOF, SLF, UF, medial lemniscus, ICP, SCP, cerebral peduncle, ALIC, PLIC, ACR, SCR, PCR, external capsule; left CCG, RIC, PTR, sagittal stratum, fornix stria terminalis, SFOF; forceps major and minor, middle cerebellar peduncle, pontine crossing tract, genu, body and splenium of CC.	7.08	(108, 80, 33)
AD	HCs < MSA-P	18619	Bilateral ATR, CST, IFOF, SLF, UF, ICP, SCP, ALIC, PLIC, RIC, ACR, SCR, PCR, External capsule, SFOF; left ILF, PTR, sagittal stratum, fornix stria terminalis; forceps minor, middle cerebellar peduncle, genu and body of CC.	7.72	(120, 120, 74)
	PD < MSA-P	34187	Bilateral ATR, CST, IFOF,SLF, UF, medial lemniscus ICP, SCP, cerebral peduncle, ALIC, PLIC, RIC, ACR, SCR, PCR, PTR, sagittal stratum, external capsule, SFOF, fornix stria terminalis; left CCG, cingulum	6.68	(118, 123, 68)

			hippocampus, ILF; right tapetum; forceps major and minor, pontine crossing tract, fornix, genu, body and splenium of CC.		
RD	HCs < MSA-P	68586	Bilateral ATR, CST, cingulum hippocampus, IFOF, ILF, SLF, UF, SLF temporal part, medial lemniscus, ICP, SCP, cerebral peduncle, ALIC, PLIC, RIC, ACR, SCR, PCR, PTR, sagittal stratum, external capsule, fornix stria terminalis, SFOF; left CCG; right tapetum; forceps major and minor, middle cerebellar peduncle, pontine crossing tract, genu, body and splenium of CC, fornix.	8.74	(121, 118, 82)
	HCs < PD	43845	Bilateral ATR, CST, IFOF, ILF, SLF, UF, SLF temporal part, ALIC, PLIC, RIC, ACR, SCR, PCR, PTR, sagittal stratum, external capsule, fornix stria terminalis, SFOF; left CCG; right tapetum; forceps major and minor, genu, body and splenium of CC.	5.14	(109, 159, 84)
	PD < MSA-P	8775	Bilateral CST, medial lemniscus, ICP, SCP; left ATR, cerebral peduncle, PLIC, SCR, external capsule; forceps minor, middle cerebellar peduncle, pontine crossing tract,	7.12	(121, 118, 82)
**NODDI**					
ICVF	HCs > MSA-P	12726	Bilateral ATR, CST, IFOF, SLF, ICP, ALIC, PLIC, ACR, SCR, external capsule, SLF, SFOF; right UF; forceps minor, middle cerebellar peduncle, genu and body of CC.	7.43	(121, 118, 81)
	HCs > PD	29579	Bilateral ATR, CST, IFOF, ILF, SLF, UF, SLF, ALIC, RIC, ACR, SCR, PTR, sagittal stratum, external capsule, SLF; left SFOF; right PCR, cingulum hippocampus, tapetum; forceps major and minor, genu and body of CC.	5.1	(108,166, 91)
	PD > MSA-P	48	Middle cerebellar peduncle.	5.95	(109, 75, 41)
ODI	HCs < MSA-P	9729	Bilateral ATR, CST, medial lemniscus, ICP, SCP cerebral peduncle, fornix stria terminalis; left cingulum hippocampus, IFOF, ILF, UF, ALIC, PLIC, RIC, PTR, sagittal stratum, external capsule; forceps major, middle cerebellar peduncle, pontine crossing tract.	6.14	(121, 135, 73)
	PD < MSA-P	87	Left external capsule.	5.95	(121,124, 84)
ISO	HCs < MSA-P	61429	Bilateral ATR, CST, CCG, cingulum hippocampus, IFOF, ILF, SLF, UF, medial lemniscus inferior cerebellar peduncle, ICP, SCP, cerebral peduncle, ALIC, PLIC, RIC, ACR, SCR, PCR, PTR, sagittal stratum, external capsule, fornix stria terminalis, SFOF; right SLF temporal part, tapetum; forceps major and minor, pontine crossing tract, fornix, genu, body, splenium of CC.	7.36	(85, 79, 30)
	PD < MSA-P	52713	Bilateral ATR, CST, CCG, cingulum hippocampus, IFOF, ILF, SLF, medial lemniscus, ICP, SCP, cerebral peduncle, ALIC, PLIC, RIC, ACR, SCR, PCR,	7.4	(85, 79, 30)

			PTR, sagittal stratum, external capsule, fornix stria terminalis; left UF, SFOF; right SLF temporal part, tapetum; forceps major and minor, middle cerebellar peduncle, pantie crossing tract, genu, body and splenium of CC, fornix		
**FWE DTI**					
FW	HCs < MSA-P	59968	Bilateral ATR, CST, CCG, cingulum hippocampus, IFOF, ILF, SLF, UF, SLF temporal part, medial lemniscus, ICP, SCP, cerebral peduncle, ALIC, PLIC, RIC, ACR SCR, PCR, PTR, sagittal stratum, external capsule, SFOF, fornix stria terminalis; right tapetum; forceps major and minor, middle cerebellar peduncle, pontine crossing tract, fornix, genu, body and splenium of CC.	6.73	(121, 119, 83)
	HCs < PD	29046	Bilateral CST, IFOF, ILF, SLF, UF, SLF temporal part, RIC, ACR, SCR, PCR, PTR, sagittal stratum, external capsule, fornix stria terminalis; left ATR; right Cingulum hippocampus, PLIC, tapetum; forceps major and minor, body and splenium of CC.	5.65	(32, 109, 75)
	PD < MSA-P	4206	Bilateral ICP, SCP; left ATR, CST, medial lemniscus, external capsule, middle cerebellar peduncle, pontine crossing tract.	5.64	(85, 79, 30)
FA_T	HCs < MSA-P	31	Left external capsule.	6.7	(116, 130, 73)
	HCs > PD	190	left ACR, ATR; forceps minor, genu of CC.	4.63	(109, 162, 82)
	PD < MSA-P	225	left IFOF, external capsule.	6.83	(116, 130, 73)
MD_T	HCs < MSA-P	3622	Bilateral ATR, CST, SLF, ALIC, PLIC, ACR, SCR, external capsule; right IFOF, SFOF; forceps minor, genu and body of CC.	7.44	(119, 117, 88)
AD_T	HCs > MSA-P	1395	Bilateral CST, SCP, cerebral peduncle; left ALIC; right ATR; middle cerebellar peduncle, pontine crossing tract.	4.74	(90, 108, 48)
RD_T	HCs < MSA-P	8587	Bilateral ATR, CST, SLF, ICP, ACR, SCR; left medial lemniscus; right IFOF, ALIC, RIC, sagittal stratum, external capsule; forceps minor, middle cerebellar peduncle, genu and body of CC.	7.02	(120, 122, 88)
	HCs < PD	11569	Bilateral ATR, IFOF, ACR, SCR, external capsule, SLF; left UF; right CST, ALIC, PLIC; forceps minor, genu and body of CC.	5.09	(81, 156, 56)
**MT-sat**					
MVF	HCs > MSA-P	63266	Bilateral ATR, CST, cingulum hippocampus, IFOF, LIF, SLF, UF, SLF temporal part, medial lemniscus, ICP, SCP, cerebral peduncle, ALIC, PLIC, RIC, ACR, SCR, PCR, PTR, sagittal stratum, external capsule, cingulum hippocampus, SFOF; right CCG, fornix stria terminalis, tapetum; forceps major and minor, middle cerebellar peduncle, pontine crossing tract, genu, body and splenium of CC, fornix.	9.27	(121, 119, 83)

PD > MSA-P	3633	Bilateral CST, medial lemniscus, ICP, SCP, middle cerebellar peduncle, pontine crossing tract.	7.27	(108, 77, 41)

Lt = left; Rt = right; ACR = anterior corona radiata; AD = axial diffusivity; AD_T = FW-corrected axial diffusivity; ALIC = anterior limb of internal capsule; ATR = anterior thalamic radiation; CC = corpus callosum; CCG = cingulum cingulate gyrus, CHp = cingulum hippocampus; CP = cerebellar peduncle; CST = cerebrospinal tract; DTI = diffusion tensor imaging; FA = fractional anisotropy; FA_T = FW-corrected fractional anisotropy; FW = free water; FWE = free water-eliminated diffusion tensor imaging; IC = internal capsule; ICP = inferior cerebellar peduncle; ICVF = intracellular volume fraction; ILF = inferior longitudinal fasciculus; ISO = isotropic volume fraction; MCP = middle cerebellar peduncle; MD = mean diffusivity; MD_T = FW-corrected mean diffusivity; MSA-P = multiple system atrophy-parkinsonian type; MT-sat = magnetization transfer-saturation; MVF = myelin volume fraction; NODDI = neurite orientation dispersion and density imaging; ODI = orientation dispersion index; PCR = posterior corona radiata; PLIC = posterior limb of internal capsule; PTR = posterior thalamic radiation; RD = radial diffusivity; RD_T = FW-corrected radial diffusivity; SCR = superior corona radiata; SCP = superior cerebellar peduncle; SFOF = superior fronto-occipital fasciculus; SLF = superior longitudinal fasciculus; SS = sagittal stratum; UF = uncinate fasciculus

Supplementary Table 3. Results of TBSS analysis for MSA-P patients, PD patients, and HCs in the second cohort

**Modality**	**Contrast**	**Cluster size**	**Anatomical region**	**Peak *T*-value**	**Peak MNI coordinates (X, Y, Z)**
**DTI**					
FA	HCs > MSA-P	45221	Bilateral ATR, CST, IFOF, ILF, SLF, UF, SLF temporal part, medial lemniscus, ICP, SCP, cerebral peduncle, PLIC, RIC, ACR, SCR, PCR, PTR, sagittal stratum, external capsule, fornix stria terminalis, SLF, UF; left ALIC; right CCG, tapetum; forceps major and minor, middle cerebellar peduncle, pontine crossing tract, genu, body, splenium of CC.	7.46	(122, 121, 80)
	PD > MSA-P	1063	Left CST, medial lemniscus, ICP; middle cerebellar peduncle, pontine crossing tract.	5.18	(99, 100, 40)
MD	HCs < MSA-P	30641	Bilateral ATR, CST, IFOF, SLF, UF, ICP, ALIC, PLIC, RIC, ACR, SCR, PCR, PTR, sagittal stratum, external capsule, SLF, SFOF; left CHp, ILF, medial lemniscus; right fornix stria terminalis; forceps major and minor, middle cerebellar peduncle, pontine crossing tract, genu, body and splenium of CC.	7.04	(116, 119 ,69)
	PD < MSA-P	11294	Bilateral CST, IFOF, SLF, medial lemniscus, ICP, ALIC, PLIC, SCR, PCR, external capsule, SLF, SFOF; left ATR, RIC, ACR; middle cerebellar peduncle, pontine crossing tract, body of CC.	6.70	(108, 93, 35)
AD	HCs < MSA-P	3036	Bilateral ATR, CST, IFOF, ALIC, PLIC, SCR, PCR, external capsule, SFOF; right SLF, ACR, SLF.	6.6	(67, 121, 68)
	PD < MSA-P	1313	Bilateral CST, ALIC, PLIC, SCR, external capsule; left ATR, SFOF.	6.85	(118, 127, 73)
RD	HCs < MSA-P	52050	Bilateral ATR, CST, IFOF, ILF, SLF, UF, SLF temporal part, medial lemniscus, ICP, SCP, ALIC, PLIC, RIC, ACR, SCR, PCR, PTR, sagittal stratum, external capsule, fornix stria terminalis, SLF; right CCG, cerebellar, tapetum; forceps major and minor, middle cerebellar peduncle, pontine crossing tract, genu, body and splenium of CC, fornix.	7.69	(122, 121, 79)
	PD < MSA-P	7078	Bilateral CST, SLF, medial lemniscus, ICP, SCR, SLF; left IFOF, SCP, PCR, external capsule; right ATR; middle cerebellar peduncle, pontine crossing tract, body of CC	6.54	(103, 97, 35)
**NODDI**					
ICVF	HCs > MSA-P	19125	Bilateral ATR, CST, IFOF, SLF, medial lemniscus, ICP, SCP, cerebral peduncle, ALIC, PLIC, RIC, ACR, SCR, PCR, external capsule, SLF, SFOF; left UF; forceps minor, middle cerebellar peduncle, genu and body of CC.	7.5	(121, 113, 82)

	PD > MSA-P	23864	Bilateral ATR, CST, IFOF, SLF, UF, medial lemniscus, ICP, SCP, cerebral peduncle, ALIC, PLIC, RIC, ACR, SCR, PCR, external capsule, SLF, SFOF; left ILF, PTR; forceps minor, middle cerebellar peduncle, genu and body of CC.	6.97	(120, 121, 88)
ODI	HCs < MSA-P	77	Bilateral external capsule, ATR, CST, SLF, medial lemniscus, ICP, SCP cerebral peduncle, SCR, SLF; right IFOF, ALIC, PLIC, RIC, ACR, PCR, external capsule; forceps minor, middle cerebellar peduncle, pontine crossing tract, genu of CC.	5.13	(123, 119, 76)
	HCs < PD	7312	Bilateral PLIC, SCR; right external capsule.	5.03	(88, 94, 57)
	PD < MSA-P	259	Left external capsule.	6.63	(64,118, 90)
ISO	HCs < MSA-P	45141	Bilateral ATR, CST, IFOF, ILF, SLF, UF, SLF temporal part, medial lemniscus, ICP, SCP, ALIC, PLIC, RIC, ACR, SCR, PCR, PTR, sagittal stratum, external capsule, fornix stria terminalis, SLF, tapetum; left CHp; right CCG, cerebral peduncle, SFOF; forceps major and minor, meddle cerebellar peduncle, pontine crossing tract, fornix, genu, body, splenium of CC.	5.96	(103, 67, 26)
	HCs < PD	49454	Bilateral ATR, CST, CHp, IFOF, ILF, SLF, UF, SLF temporal part, cerebral peduncle, ALIC, PLIC, RIC, ACR, SCR, PCR, PTR, sagittal stratum, external capsule, fornix stria terminalis, SLF, tapetum; left SFOF, UF; right SCP; forceps major and minor, genu, body and splenium of CC.	5.36	(52, 56, 97)
**FWE DTI**					
FW	HCs < MSA-P	49090	Bilateral ATR, CST, CCG, IFOF, ILF, SLF, UF, SLF temporal part, ALIC, PLIC, RIC, ACR, SCR, PCR, PTR, sagittal stratum, external capsule, SLF, tapetum; right cerebral peduncle, fornix stria terminalis, SFOF; forceps major and minor, middle cerebellar peduncle, pontine crossing tract, genu, body and splenium of CC.	6.52	(127, 129, 113)
	HCs < PD	44056	Bilateral ATR, CST, IFOF, ILF, SLF, UF, SLF temporal part, cerebral peduncle, ALIC, PLIC, RIC, ACR, SCR, PCR, PTR, sagittal stratum, external capsule, fornix stria terminalis, SLF, tapetum; left CHp; right SCP; forceps major and minor, genu, body and splenium of CC.	5.11	(111, 144, 58)
FA_T	HCs < MSA-P	2820	Bilateral CST, medial lemniscus, ICP, SCP, SCR; Left cerebral peduncle; right ATR, external capsule; middle cerebellar peduncle, pontine crossing tract.	6.2	(58, 123, 83)
	PD < MSA-P	2074	Bilateral CST, ICP; right medial lemniscus SCP; middle cerebellar peduncle, pontine crossing tract.	5.32	(105, 91, 35)

MD_T	HCs < MSA-P	16376	Bilateral ATR, CST, IFOF, SLF, medial lemniscus, ICP, SCP, ALIC, PLIC, ACR, SCR, PCR, external capsule, SLF, SFOF; left cerebral peduncle, RIC; right UF; middle cerebellar peduncle, pontine crossing tract, genu and body of CC.	7.69	(120, 113, 87)
	PD < MSA-P	27862	Bilateral ATR, CST, IFOF, SLF, UF, medial lemniscus, ICP, SCP, cerebral peduncle, ALIC, PLIC, RIC, ACR, SCR, PCR, external capsule, SLF, SFOF; right CCG; forceps minor, middle cerebellar peduncle, pontine crossing tract, body and splenium of CC	7.34	(61, 124, 91)
AD_T	HCs > MSA-P	94	right ALIC, PLIC, SCR, external capsule.	5.42	(63, 118, 90)
	HCs > PD	180	right CST.	4.13	(68, 92, 118)
	PD < MSA-P	6219	Bilateral ATR, CST, medial lemniscus, SCP, cerebral peduncle, ALIC, PLIC, ACR, SCR, external capsule, SFOF; left ICP; right IFOF, SLF, RIC, PCR, SLF; middle cerebellar peduncle.	7.67	(64, 120, 97)
RD_T	HCs < MSA-P	22127	Bilateral ATR, CST, IFOF, SLF, UF, medial lemniscus, ICP, SCP, cerebral peduncle, PLIC, RIC, ACR, SCR, PCR, external capsule, SLF; left ALIC, fornix stria terminalis, SFOF; forceps major and minor, middle cerebellar peduncle, pontine crossing tract, genu, body and splenium of CC.	7.64	(120, 118, 88)
	PD < MSA-P	6892	Bilateral CTR, SFOF, medial lemniscus, ICP, SCR, PCR, SLF; right ATR, IFOF, UF, SCP, ACR, external capsule; middle cerebellar peduncle, pontine crossing tract, body of CC.	7.03	(63, 117, 107)

Lt = left; Rt = right; ACR = anterior corona radiata; AD = axial diffusivity; AD_T = FW-corrected axial diffusivity; ALIC = anterior limb of internal capsule; ATR = anterior thalamic radiation; CC = corpus callosum; CCG = cingulum cingulate gyrus; CHp = cingulum hippocampus; CP = cerebellar peduncle; CST = cerebrospinal tract; DTI = diffusion tensor imaging; FA = fractional anisotropy; FA_T = FW-corrected fractional anisotropy; FW = free water; FWE = free water-eliminated diffusion tensor imaging; IC = internal capsule; ICP = inferior cerebellar peduncle; ICVF = intracellular volume fraction; IFOF = inferior front occipital fasciculus; ILF = inferior longitudinal fasciculus; ISO = isotropic volume fraction; MCP = middle cerebellar peduncle; MD = mean diffusivity; MD_T = FW-corrected mean diffusivity; MSA-P = multiple system atrophy-parkinsonian type; MT-sat = magnetization transfer-saturation; MVF = myelin volume fraction; NODDI = neurite orientation dispersion and density imaging; ODI = orientation dispersion index; PCR = posterior corona radiata; PLIC = posterior limb of internal capsule; PTR = posterior thalamic radiation; RD = radial diffusivity; RD_T = FW-corrected radial diffusivity; RIC = Retrolenticular part of internal capsule; SCR = superior corona radiata; SCP = superior cerebellar peduncle; SFOF = superior fronto-occipital fasciculus; SLF = superior longitudinal fasciculus; SS = sagittal stratum; UF = uncinate fasciculus

Supplementary Table 4. White matter and nigral regions and parameters showing a significant difference in the region-of-interest (ROI) analysis

		First cohort			Second cohort		
		**HCs**	**PD**	**MSA-P**	**HCs**	**PD**	**MSA-P**
**White matter**							
Middle cerebellar peduncle	**ICVF**	0.794 ± 0.022 [a]	0.790 ± 0.026 [a]	0.727 ± 0.063 [b,c]	0.743 ± 0.020 [a,b]	0.748 ± 0.018 [c]	0.677 ± 0.079 [c]
	**FW**	0.180 ± 0.009 [a]	0.183 ± 0.008 [a]	0.198 ± 0.015 [b,c]	0.184 ± 0.012 [a,b]	0.183 ± 0.012 [c]	0.203 ± 0.018 [c]
	**RD_T**	0.00020 ± 0.00001 [a]	0.00020 ± 0.00001 [a]	0.00023 ±0.00003 [b,c]	0.00022 ± 0.000010 [a,b]	0.00022 ± 0.000012 [c]	0.00027 ± 0.000054 [c]
	**MVF**	0.333 ± 0.014 [a]	0.324 ± 0.019 [a]	0.296 ± 0.022 [b,c]			
Inferior cerebellar peduncle	**ICVF**	0.723 ± 0.028 [a]	0.721 ± 0.029 [a]	0.681 ± 0.045 [b,c]	0.640 ± 0.016 [a,b]	0.648 ± 0.019 [c]	0.602 ± 0.045 [c]
	**FW**	0.207 ± 0.020 [a]	0.208 ± 0.013 [a]	0.230 ± 0.020 [b,c]	0.201 ± 0.020	0.203 ± 0.019	0.206 ± 0.025
	**MVF**	0.287 ± 0.013 [a]	0.279 ± 0.015 [a]	0.263 ± 0.018 [b,c]			
Pontine crossing tract	**ICVF**	0.806 ± 0.029	0.822 ± 0.043	0.780 ± 0.068	0.701 ± 0.029	0.710 ± 0.026	0.655 ± 0.081
	**FW**	0.203 ± 0.023	0.209 ± 0.018	0.218 ± 0.027	0.208 ± 0.013 [a,b]	0.210 ± 0.019 [c]	0.239 ± 0.027 [c]
	**MVF**	0.311 ± 0.017 [a]	0.312 ± 0.014 [a]	0.288 ± 0.018 [b,c]			
Corticospinal tract	**ICVF**	0.713 ± 0.017	0.708 ± 0.023	0.689 ± 0.027	0.713 ± 0.017 [a,b]	0.716 ± 0.021 [c]	0.684 ± 0.027 [c]
	**FW**	0.193 ± 0.007 [a]	0.197 ± 0.008 [a]	0.205 ± 0.009 [b,c]	0.204 ± 0.025 [a]	0.215 ± 0.028	0.231 ± 0.034 [a]
	**MVF**	0.356 ± 0.013 [a]	0.349 ± 0.016	0.336 ± 0.012 [c]			
**Substantia nigra pars compacta**							
Anterior part of SNc	**ICVF**	0.724 ± 0.057 [a,b]	0.768 ± 0.065 [c]	0.773 ± 0.06 [c]	0.722 ± 0.059 [b]	0.759 ± 0.055 [c]	0.748 ± 0.039
	**FW**	0.244 ± 0.045 [b]	0.285 ± 0.063 [c]	0.268 ± 0.052	0.255 ± 0.056	0.258 ± 0.057	0.26 ± 0.072
	**FA_T**	0.58 ± 0.102 [a,b]	0.67 ± 0.079 [c]	0.697 ± 0.08 [c]	0.595 ± 0.079 [a,b]	0.66 ± 0.088 [c]	0.678 ± 0.087 [c]
	**RD_T**	0.00025 ± 0.00006 [a,b]	0.0002 ± 0.00006 [c]	0.00018 ± 0.00005 [c]	0.00025 ± 0.00007 [b]	0.0002 ± 0.00006 [c]	0.00021 ± 0.00007
Posterior part of SNc	**ICVF**	0.693 ± 0.062 [a,b]	0.733 ± 0.04 [c]	0.736 ± 0.062 [c]	0.654 ± 0.068	0.694 ± 0.079	0.682 ± 0.081
	**FW**	0.247 ± 0.041 [a,b]	0.287 ± 0.032 [c]	0.271 ± 0.035 [c]	0.188 ± 0.037 [b]	0.216 ± 0.036 [c]	0.192 ± 0.056
	**FA_T**	0.526 ± 0.046 [a,b]	0.612 ± 0.049 [a,c]	0.673 ± 0.062 [c]	0.521 ± 0.068 [a,b]	0.594 ± 0.103 [c]	0.63 ± 0.099 [c]
	**RD_T**	0.00029 ± 0.00004 [a,b]	0.00024 ± 0.00004 [c]	0.00023 ± 0.00005 [c]	0.00031 ± 0.00006 [a,b]	0.00026 ± 0.00008 [c]	0.00025 ± 0.00008 [c]

FW, free-water; HCs, healthy controls; ICVF, intracellular volume fraction; MVF, myelin volume fraction; MSA-P, multiple system atrophy-parkinsonian type; PD, Parkinson's disease; RD_T, FW-corrected radial diffusivity;

$P < 0.05$ (false discovery rate [FDR] corrected); [a] versus MSA-P, [b] versus Parkinson's disease, [c] versus healthy controls

Supplementary Table 5. Stepwise logistic regression analysis and ROC study results of two cohorts

	Stepwise - first cohort*					ROC study - first cohort							ROC study - second cohort					
	Step	Region	Action	R^2		Cut-off value	AUC	R^2	P value	Sp	Se		Cut-off value	AUC	R^2	P value	Sp	Se
**ICVF**	1	MCP	Add	0.27		0.731	0.79	0.27	<0.001	100.0	57.1		0.710	0.82	0.39	<0.01	100.0	70.0
	2	SLF	Add	0.42		0.564	0.53	0.01	0.04	47.4	71.4		0.639	0.75	0.13	0.03	58.8	90.0
	3	IFOF	Add	0.50		0.590	0.76	0.12	0.01	73.7	81.0		0.555	0.67	0.05	0.19	58.8	80.0
	4	CST	Add	0.63		0.687	0.70	0.10	0.02	84.2	52.4		0.693	0.72	0.18	0.01	100.0	50.0
					Combined	**MCP 0.712**<br>**SLF 0.573**<br>**IFOF 0.602**<br>**CST 0.712**	**0.96**	**0.63**	**<0.001**	**94.7**	**85.7**	Combined	**MCP 0.751**<br>**SLF 0.632**<br>**IFOF 0.552**<br>**CST 0.764**	**0.93**	**0.50**	**0.0014**	**94.1**	**80.0**
**FW**	1	ICP	Add	0.26		0.216	0.84	0.26	<0.01	79.0	91.0		0.224	0.49	0.01	0.74	88.2	40.0
	2	ILF	Add	0.36		0.210	0.62	0.04	0.05	68.4	66.7		0.199	0.59	0.01	0.89	52.9	70.0
	3	MCP	Add	0.44		0.186	0.80	0.25	<0.01	68.4	85.7		0.186	0.84	0.28	0.01	70.6	90.0
	4	SLF	Add	0.64		0.193	0.59	0.01	0.04	57.9	76.2		0.208	0.64	0.07	0.11	100.0	30.0
					Combined	**ICP 0.209**<br>**ILF 0.207**<br>**MCP 0.172**<br>**SLF 0.203**	**0.96**	**0.64**	**<0.001**	**94.7**	**95.2**	Combined	**ICP 0.224**<br>**ILF 0.223**<br>**MCP 0.200**<br>**SLF 0.214**	**0.92**	**0.47**	**0.0021**	**88.2**	**90.0**
**MVF**	1	MCP	Add	0.2888		0.321	0.84	0.29	<0.001	63.2	95.2		-	-	-	-	-	-
	2	EC	Add	0.2904		0.301	0.68	0.08	0.03	42.1	90.5		-	-	-	-	-	-
	3	SLF	Add	0.4131		0.411	0.45	0.01	0.03	21.1	90.5		-	-	-	-	-	-
	4	UF	Add	1.000		0.319	0.53	0.01	0.35	79.0	33.3		-	-	-	-	-	-
					Combined	**MCP 0.303**<br>**EC 0.284**<br>**SLF 0.409**<br>**UF 0.296**	**1.00**	**1.00**	**<0.001**	**100.0**	**100.0**		-	-	-	-	-	-

*The forward stepwise analysis was set to p <0.05 to include the item.

AUC = area under curve; CST = corticospinal tract; EC = external capsule; FW = free water; ICVF = intracellular volume fraction; ICP = inferior cerebellar peduncle; ILF, inferior longitudinal fasciculus; ILOF, inferior fronto-occipital fasciculus; MCP = middle cerebellar peduncle; MVF = myelin volume fraction; ROC, receiver operating characteristic; Se = sensitivity; Sp, specificity; SLF, superior longitudinal fasciculus

Supplementary Table 6. Acquisition Parameters used for the first and second cohorts

	**DWI**	
	**First Cohort**	**Second Cohort**
TR / TE (ms)	3300 / 70	3600 / 79
Field of View (mm)	229 × 229	204 × 204
Matrix size	130 × 130	120 × 120
Resolution (mm)	1.8 × 1.8	1.7 × 1.7
Slice thickness (mm)	1.8	1.7
Acquisition time (min)	07.29	07.04
b-values (s / mm^2)	0 / 1000 / 2000	0 / 700 / 2000
Volume(s) / Gradient directions/ Gradient directions	1 / 64 / 64	17 / 40 / 80
Flip angle (°)	90	90

DWI = diffusion weighted imaging; TE = echo time; TI = inversion time; TR = repetition time.

Supplementary Figure 1. The actual head angle calculation

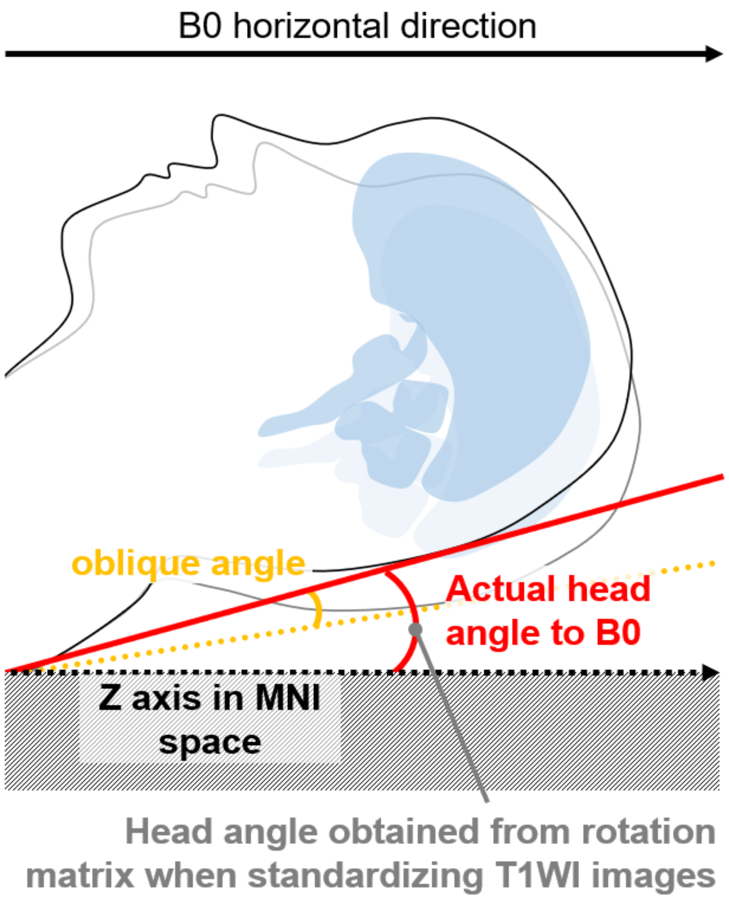

The actual head angle was measured by correcting the oblique angle at the imaging from DICOM header information and calculating the angle on the image from the rotation matrix when standardizing T1-WI images. The calculated data was confirmed that there was no statistically significant difference ($p > 0.05$) between the disease groups.

Supplementary Method 1. Measuring method of substantia nigra pars compacta (SNc) with a square- region-of-interest (ROI)

Method

The SNc ROI was manually created using ITK-SNAP[1] to prevent the effects of measurements from adjacent structures. ROIs of the anterior substantia nigra (aSN) and the posterior substantia nigra (pSN) were set on b=0 images adjusted to standard space according to the following method. ROIs consisting of four 1 × 1-mm pixels arranged in a square were set at four locations for both aSN and pSN on two consecutive slices (Fig. 1).

1) First, the level at which the substantia nigra and red nucleus of the midbrain were fully visible was identified.

2) The analyst moved to the caudal side of 3 to 4 slices (slice thickness 1 mm) and selected a level where the red nucleus was not visible or only slightly visible.[2]

3) Two-by-two-pixel squares were placed on the front, back, left, and right in the inner boundary area of the region showing low signal.

4) Four similar squares were arranged in the cross section on the caudal side of one continuous slice.

5) To ensure the accuracy of the ROIs, the same evaluations was performed by two raters and the ICCs were calculated.[3]

6) The mean value of the two raters was used for the statistical evaluation of the ROIs.

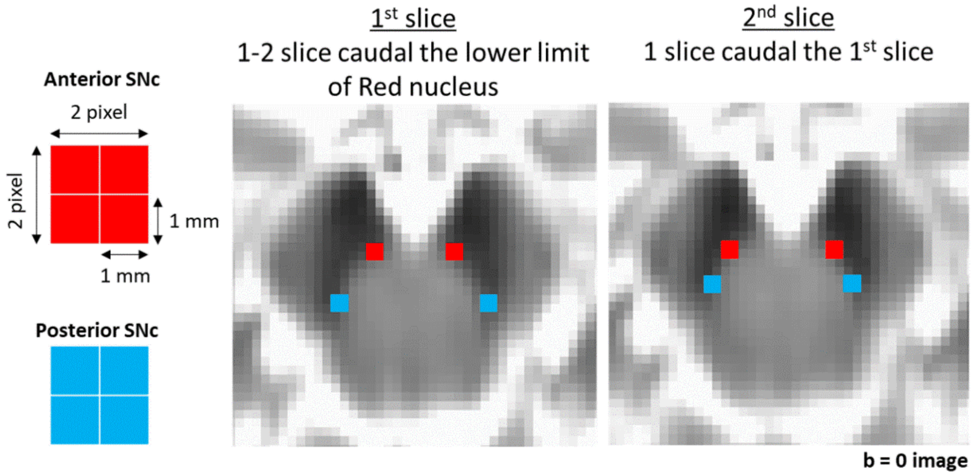

FIG. The anterior and posterior ROI of substantia nigra. The SNc ROI was manually created with using b=0 images adjusted to standard space. Two-by-two-pixel squares were placed on the front, back, left, and right in the inner boundary area of the region showing low signal. Four similar squares were arranged in the cross section on the caudal side of one continuous slice (see Methods).

Results

ROIs were manually placed in the anterior (aSN) and posterior parts (pSN) of the SNc, and the mean values of the two raters were compared between the subject groups. The intraclass correlation coefficient (ICC) between the two raters for each set of ROIs averaged 0.97 (range 0.94–0.99).

In the first cohort, there was significantly higher FW and FA_T in the aSN of the MSA-P patients compared with the HCs, whereas the pSN showed significantly higher ICVF, FW, and FA_T, and significantly lower RD_T in the MSA-P and PD patients compared with the HCs. Similar results were observed in the second cohort, except for FA_T in the aSN.

Discussion

Correlation results of square-ROI and AAL3 [4] showed that ICVF, FW, FA_T, and RD_T all had a significant positive correlation, and the correlation coefficient was 0.40 to 0.84. The results of the three-group comparison analyzed using square-ROI were in agreement with the comparison results of AAL3 in all tendencies. Therefore, it is possible that two square ROIs in the front, back, left, and right can represent SN degeneration.

Reference

1 Yushkevich, P. A. *et al.* User-guided 3D active contour segmentation of anatomical structures: significantly improved efficiency and reliability. *Neuroimage* **31**, 1116-1128, doi:10.1016/j.neuroimage.2006.01.015 (2006).

2 Vaillancourt, D. E. *et al.* High-resolution diffusion tensor imaging in the substantia nigra of de novo Parkinson disease. *Neurology* **72**, 1378-1384, doi:10.1212/01.wnl.0000340982.01727.6e (2009).

3 Shrout, P. E. & Fleiss, J. L. Intraclass correlations: uses in assessing rater reliability. *Psychol Bull* **86**, 420-428, doi:10.1037//0033-2909.86.2.420 (1979).

4 Rolls, E. T., Huang, C. C., Lin, C. P., Feng, J. & Joliot, M. Automated anatomical labelling atlas 3. *Neuroimage* **206**, 116189, doi:10.1016/j.neuroimage.2019.116189 (2020).
