# Supplementary material for: White matter and nigral alterations in multiple system atrophy-parkinsonian type
Source: NPJ Parkinsons Dis. 2021 Oct 29;7:96. doi: 10.1038/s41531-021-00236-0 (PMC8556415; doi:10.1038/s41531-021-00236-0)
Supplement: Supplementary file 2 — Reporting Summary [file 41531_2021_236_MOESM2_ESM.pdf]

nature portfolio

Corresponding author(s): Taku Hatano

Last updated by author(s): 2021-Aug-07

Reporting Summary

Nature Portfolio wishes to improve the reproducibility of the work that we publish. This form provides structure for consistency and transparency in reporting. For further information on Nature Portfolio policies, see our Editorial Policies and the Editorial Policy Checklist.

Statistics

For all statistical analyses, confirm that the following items are present in the figure legend, table legend, main text, or Methods section.

n/a	Confirmed	
☐	☒	The exact sample size (n) for each experimental group/condition, given as a discrete number and unit of measurement
☐	☒	A statement on whether measurements were taken from distinct samples or whether the same sample was measured repeatedly
☐	☒	The statistical test(s) used AND whether they are one- or two-sided<br>*Only common tests should be described solely by name; describe more complex techniques in the Methods section.*
☐	☒	A description of all covariates tested
☐	☒	A description of any assumptions or corrections, such as tests of normality and adjustment for multiple comparisons
☐	☒	A full description of the statistical parameters including central tendency (e.g. means) or other basic estimates (e.g. regression coefficient) AND variation (e.g. standard deviation) or associated estimates of uncertainty (e.g. confidence intervals)
☐	☒	For null hypothesis testing, the test statistic (e.g. F, t, r) with confidence intervals, effect sizes, degrees of freedom and P value noted<br>*Give P values as exact values whenever suitable.*
☒	☐	For Bayesian analysis, information on the choice of priors and Markov chain Monte Carlo settings
☒	☐	For hierarchical and complex designs, identification of the appropriate level for tests and full reporting of outcomes
☒	☐	Estimates of effect sizes (e.g. Cohen's d, Pearson's r), indicating how they were calculated

Our web collection on statistics for biologists contains articles on many of the points above.

Software and code

Policy information about availability of computer code

Data collection	The tools used for MRI pre-processing in this paper are available from the following; EDDY, TOPUP and DTIFIT toolboxes (FSL, FMRIB Software Library; http://www.fmrib.ox.ac.uk/fsl), Matlab Toolbox5 (http://www.nitrc.org/projects/noddi_toolbox), AMICO (Accelerated Microstructure Imaging via Convex Optimization), and Matlab (MathWorks, Natick, MA, USA). The FMRIB nonlinear registration tool used for TBSS is available from FSL (http://www.fmrib.ox.ac.uk/fsl).
Data analysis	JMP v14; SAS Inc., Cary, NC, USA; or the FSL package for the general linear model analysis

For manuscripts utilizing custom algorithms or software that are central to the research but not yet described in published literature, software must be made available to editors and reviewers. We strongly encourage code deposition in a community repository (e.g. GitHub). See the Nature Portfolio guidelines for submitting code & software for further information.

Data

Policy information about availability of data

All manuscripts must include a data availability statement. This statement should provide the following information, where applicable:

- Accession codes, unique identifiers, or web links for publicly available datasets
- A description of any restrictions on data availability
- For clinical datasets or third party data, please ensure that the statement adheres to our policy

The data that support the findings of this study are available from the corresponding author, TH, upon reasonable request.

Field-specific reporting

Please select the one below that is the best fit for your research. If you are not sure, read the appropriate sections before making your selection.

☒ Life sciences ☐ Behavioural & social sciences ☐ Ecological, evolutionary & environmental sciences

For a reference copy of the document with all sections, see nature.com/documents/nr-reporting-summary-flat.pdf

Life sciences study design

All studies must disclose on these points even when the disclosure is negative.

Sample size	This study is a case-control study, thus no sample size was calculation.
Data exclusions	Cases with cognitive decline based on Mini-mental state exam and minimal vascular lesions based on MRI were excluded from the study.
Replication	We confirmed the replication of the data.
Randomization	N/A
Blinding	N/A

Reporting for specific materials, systems and methods

We require information from authors about some types of materials, experimental systems and methods used in many studies. Here, indicate whether each material, system or method listed is relevant to your study. If you are not sure if a list item applies to your research, read the appropriate section before selecting a response.

Materials & experimental systems

n/a	Involved in the study	
☒	☐	Antibodies
☒	☐	Eukaryotic cell lines
☒	☐	Palaeontology and archaeology
☒	☐	Animals and other organisms
☒	☐	Human research participants
☐	☒	Clinical data
☒	☐	Dual use research of concern

Methods

n/a	Involved in the study	
☒	☐	ChIP-seq
☒	☐	Flow cytometry
☐	☒	MRI-based neuroimaging

Clinical data

Policy information about clinical studies

All manuscripts should comply with the ICMJE guidelines for publication of clinical research and a completed CONSORT checklist must be included with all submissions.

Clinical trial registration	We received ethical approval from Juntendo University (14-011).
Study protocol	Age- and sex-matched patients with MSA-P (n=21, n=10 first and second cohorts, respectively), Parkinson's disease patients (n=19, 17), and healthy controls (n=20, 25) were enrolled. Magnetization transfer saturation imaging (MT-sat) and dMRI were obtained using 3-T MRI. Measurements obtained from diffusion tensor imaging (DTI), free water elimination DTI, neurite orientation dispersion and density imaging (NODDI), and MT-sat were compared between groups.
Data collection	Clinical data were carefully evaluated by three movement disorder specialists (T.O., T.H., and H.T.A.), and cases with apparent cognitive impairment based on the mini-mental state examination.
Outcomes	Tract-based spatial statistics analysis revealed differences in diffuse white matter alterations in the free-water fractional volume, myelin volume fraction, and intracellular volume fraction between patients with MSA-P and healthy controls, whereas free-water and MT-sat differences were limited to the middle cerebellar peduncle in comparison with those with Parkinson's disease. Region-of-interest analysis of white matter and SNc revealed significant differences in the middle and inferior cerebellar peduncle, pontine crossing tract, corticospinal tract, and SNc between MSA-P and healthy controls and/or Parkinson's disease patients.

Magnetic resonance imaging

Experimental design

Design type	Diffusion MRI and Myelin sensitive imaging, brain scan
Design specifications	Total scan time was about 60 min for each subject
Behavioral performance measures	N/A

Acquisition

Imaging type(s)	Structural and diffusion MRI
Field strength	3T-MRI scanner (MAGNETOM Prisma, Siemens Healthcare, Erlangen, Germany)
Sequence & imaging parameters	1. First cohort: all participants<br>Multi-shell DWI was performed using a spin-echo echo planar imaging sequence, which included two b values of 1000 and 2000 s/mm2 in the first cohort.<br>The sequence parameters used for the first cohort were TR = 3300 ms, TE = 70 ms, field of view = 229 ? 229 mm, matrix size = 130 x 130, resolution = 1.8 x 1.8 mm, slice thickness = 1.6 mm, and acquisition time = 07.29 min.<br>The sequences for the MT-sat were set as follows: for MT-off and MT-on scanning, TR = 24 ms, TE = 2.53 ms, flip angle = 5°; for T1-WI, TR = 10 ms; TE = 2.53 ms, flip angle = 13°, with parallel imaging using GeneRalized Autocalibrating Partially Parallel Acquisitions with a factor of 2 in the phase-encoding direction, 7/8 partial Fourier acquisition in the partition direction, bandwidth = 260 Hz/pixel, field of view = 224 × 224 mm, matrix = 128 × 128, slice thickness = 1.8 mm, and acquisition time = 6 min 25 s.<br><br>2. Second cohort: all participants<br>Multi-shell DWI was performed using a spin-echo echo planar imaging sequence, which included two b values of 700 and 2000 s/mm2 in the second cohort.<br>The sequence parameters used for the second cohort were TR = 3600 ms, TE = 79 ms, field of view = 204 × 204 mm, matrix size = 120 × 120, resolution = 1.7 × 1.7 mm, slice thickness = 1.7 mm, and acquisition time = 7.04 min.
Area of acquisition	Whole brain scan was performed.

Diffusion MRI ☒ Used ☐ Not used

Parameters

1. First cohort: all participants
Multi-shell DWI was performed using a spin-echo echo planar imaging sequence, which included two b values of 1000 and 2000 s/mm2 in the first cohort.
DTI measures were obtained using an ordinary least square method applied to the DWI with b=0 and 1000 s/mm2. FA, MD, AD, and RD maps were calculated using the DTIFIT tool implemented in FSL (FMRIB Software Library 5.0.9; Oxford Centre for Functional MRI of the Brain, UK; www.fmrib.ox.ac.uk/fsl), which is based on standard formulae.
The NODDI model was applied to the MRI results using the NODDI Matlab Toolbox5 (http://www.nitrc.org/projects/noddi_toolbox). ICVF, ODI, and ISOVF maps were calculated using AMICO (Accelerated Microstructure Imaging via Convex Optimization). FWE-DTI was processed using a regularized bi-tensor model in Matlab (MathWorks, Natick, MA, USA), and FAT, MDT, RDT, ADT, and FW maps were calculated. ISOVF and FW, obtained from NODDI and FWE-DTI, respectively, are both indicators of extracellular free-water content in the brain.
MT-sat was calculated using an in-house MATLAB script based on previously described theory. MVF maps (only calculated for the first cohort) were obtained using an MT-sat correction factor of 0.1.

2. Second cohort: all participants
Multi-shell DWI was performed using a spin-echo echo planar imaging sequence, which included two b values of 700 and 2000 s/mm2 in the second cohort.
Similar to the first cohort, DTI, NODDI and FWE-DTI were calculated. However, MT-sat was not acquired, so MVF was not calculated.

Preprocessing

Preprocessing software	The EDDY and TOPUP toolboxes were used to correct susceptibility-induced geometric distortions, eddy current distortions, and inter-volume subject motion in the DWI datasets. FMRIB nonlinear registration tool were used for tract-based spatial statistics analysis (TBSS) analysis. ITK-SNAP was used for analysis region-of-interest of substantia nigra pars compacta.
Normalization	We evaluated participants' white matter alterations using a tract-based spatial statistics analysis (TBSS) skeleton projection step. Non-liner registration was used in this step.
Normalization template	Montreal Neurological Institute (152; MNI) space was used for tract-based spatial statistics analysis (TBSS) analysis.
Noise and artifact removal	The EDDY and TOPUP toolboxes were used to correct susceptibility-induced geometric distortions, eddy current distortions, and inter-volume subject motion in the DWI datasets. All DWI datasets were then visually checked in the axial, sagittal, and coronal views, and were confirmed to be free from severe artifacts such as gross geometric distortion, signal dropout, and bulk motion.

Volume censoring

Exclude peripheral tracts and gray matter on TBSS analysis.

Statistical modeling & inference

Model type and settings

1. Participants background: The background demographics include continuous variables and nominal variables.
2. White matter: TBSS analysis and ROI analysis were performed. White matter was evaluated using automated ROI analysis. Maps showing significant clusters on TBSS analyses were localized using the John Hopkins University (JHU) white matter tractography atlas and the International Consortium of Brain Mapping (ICBM)-DTI-81 white matter atlas. Thirty ROIs were selected based on the JHU and ICBM atlases and were used to compare overall white matter differences.
3. Substantia nigra pars compacta (SNc): ROI analysis was performed. The SNc ROI was manually created.

Effect(s) tested

1. Participants background: Wilcoxon analysis or Kruskal-Wallis analysis was used for continuous variables and the Pearson Chi-square test was also used for nominal variables.
2&3. White matter and SNc: Pairwise comparisons between groups were performed using nonlinear regression analysis in TBSS analysis of white matter. For each ROI, Kruskal-Wallis analysis was performed for group comparisons, and the results were corrected for multiple tests using the Benjamini-Hochberg FDR method. Post-hoc analyses of NODDI, FWE-DTI, and MT-sat measures were calculated using Steel-Dwass analysis. Spearman's rank correlation was used to examine correlations between MRI parameters within regions that showed significant differences in the ROI analyses.

Specify type of analysis: ☐ Whole brain ☐ ROI-based ☒ Both

Anatomical location(s)

1. Whole brain: Montreal Neurological Institute (152; MNI) space and John Hopkins University (JHU) white matter tractography atlas and the International Consortium of Brain Mapping (ICBM)-DTI-81 white matter atlas.
2. ROI-based:
white matter: using the John Hopkins University (JHU) white matter tractography atlas and the International Consortium of Brain Mapping (ICBM)-DTI-81 white matter atlas.
SNc: manually created

Statistic type for inference
(See Eklund et al. 2016)

Tract-based spatial statistics analysis (TBSS) (one of cluster analysis) were performed for white matter analysis, no fMRI data acquired.

Correction

TBSS analysis was corrected p-value with family-wise error. Other analysis were corrected p-value with Benjamini-Hochberg FDR method.

Models & analysis

n/a	Involved in the study
☒	☐ Functional and/or effective connectivity
☒	☐ Graph analysis
☐	☒ Multivariate modeling or predictive analysis

Multivariate modeling and predictive analysis

Nominal logistic analysis was also performed to differentiate diseases according to an ROC curve for the ROIs, and was followed by stepwise regression analysis.
